# Enhanced medical diagnosis for dOCTors: a perspective of optical coherence tomography

**DOI:** 10.1117/1.JBO.26.10.100601

**Published:** 2021-10-20

**Authors:** Rainer Leitgeb, Fabian Placzek, Elisabet Rank, Lisa Krainz, Richard Haindl, Qian Li, Mengyang Liu, Marco Andreana, Angelika Unterhuber, Tilman Schmoll, Wolfgang Drexler

**Affiliations:** a Medical University of Vienna, Center for Medical Physics and Biomedical Engineering, Vienna, Austria; b Medical University of Vienna, Christian Doppler Laboratory OPTRAMED, Vienna, Austria; c Carl Zeiss Meditec, Inc., Dublin, California, United States

**Keywords:** optical coherence tomography, multimodal OCT, multimodal OCT endoscopy, miniaturized OCT, non-linear optical microscopy, photoacoustic imaging, functional OCT, contrast enhanced OCT, optical coherence elastography, OCT angiography, artificial intelligence enhanced OCT

## Abstract

**Significance:** After three decades, more than 75,000 publications, tens of companies being involved in its commercialization, and a global market perspective of about USD 1.5 billion in 2023, optical coherence tomography (OCT) has become one of the fastest successfully translated imaging techniques with substantial clinical and economic impacts and acceptance.

**Aim:** Our perspective focuses on disruptive forward-looking innovations and key technologies to further boost OCT performance and therefore enable significantly enhanced medical diagnosis.

**Approach:** A comprehensive review of state-of-the-art accomplishments in OCT has been performed.

**Results:** The most disruptive future OCT innovations include imaging resolution and speed (single-beam raster scanning versus parallelization) improvement, new implementations for dual modality or even multimodality systems, and using endogenous or exogenous contrast in these hybrid OCT systems targeting molecular and metabolic imaging. Aside from OCT angiography, no other functional or contrast enhancing OCT extension has accomplished comparable clinical and commercial impacts. Some more recently developed extensions, e.g., optical coherence elastography, dynamic contrast OCT, optoretinography, and artificial intelligence enhanced OCT are also considered with high potential for the future. In addition, OCT miniaturization for portable, compact, handheld, and/or cost-effective capsule-based OCT applications, home-OCT, and self-OCT systems based on micro-optic assemblies or photonic integrated circuits will revolutionize new applications and availability in the near future. Finally, clinical translation of OCT including medical device regulatory challenges will continue to be absolutely essential.

**Conclusions:** With its exquisite non-invasive, micrometer resolution depth sectioning capability, OCT has especially revolutionized ophthalmic diagnosis and hence is the fastest adopted imaging technology in the history of ophthalmology. Nonetheless, OCT has not been completely exploited and has substantial growth potential—in academics as well as in industry. This applies not only to the ophthalmic application field, but also especially to the original motivation of OCT to enable optical biopsy, i.e., the *in situ* imaging of tissue microstructure with a resolution approaching that of histology but without the need for tissue excision.

## 1 Introduction

Optical coherence tomography (OCT) is one of the most innovative and successfully translated imaging techniques with substantial clinical and economic impacts and acceptance.^1^^,^^2^ OCT is a non-invasive optical analog to ultrasound (US) with significantly higher resolution (<1  μm) enabling three- and four-dimensional high-speed (>millions of A-scans/s) imaging with tissue penetration of up to 2 mm, closely matching that of conventional histopathology. The year 2021 marks not only the 30th birthday of OCT (assuming its initiation with the *Science* paper by Huang et al.^3^ in 1991) but also the 35th birthday of low-coherence interferometry and optical ranging in biological systems.^4^^,^^5^ In the last three decades, more than 75,000 OCT related papers have been published (about two thirds in ophthalmology) with continuous yearly increases of published articles.^6^ Breaking through the 1000 publications/year barrier was initiated in 2005/2006 with the introduction of spectral domain OCT (SD OCT). In 2020, the OCT-related scientific output was more than 7800 papers, resulting in nearly one paper every single hour on every single day of the year. Extrapolating this publishing performance, a saturation of yearly publication output at about 9500 can be expected around 2030. After 30 years, it is interesting and important to benchmark this performance with other medical imaging techniques:^6^ multiphoton microscopy (MPM) [including second harmonic generation (SHG) and third harmonic generation (THG)], developed about three decades before OCT,^7^^,^^8^ has about 50,000 publications so far; photoacoustic imaging (PAI), established in the 1970s,^9^^,^^10^ has about 15,000 papers; and confocal microscopy, developed in the 1940s,^11^^,^^12^ has about 145,000. Developed in the 1940s,^13^ US imaging has contributed to about 160,000 papers; positron emission tomography (PET), initiated in the 1970s,^14^^,^^15^ has about 175,000; computed tomography (CT), developed in the 1930s,^16^ has about 750,000; and magnetic resonance imaging (MRI), developed in the late 1940s,^17^ has close to 1,000,000 publications. This dominance in publications of radiology and nuclear medicine imaging technologies is also one of the reasons why medical imaging is, in general, associated with MRI, CT, PET, or US. It is important to note, though, that from a medical imaging market perspective, optical imaging technologies dominate with 66% versus 34% for radiology and nuclear medicine imaging technologies. In addition, in the United States alone, about 450,000 physicians use primarily optical imaging techniques; 60,000 use primarily radiologic imaging; and about 130,000 use both.^18^

In the last three decades, OCT has revolutionized ophthalmic diagnosis, therapy monitoring, and guidance. Every second, a human gets a retinal OCT scan; therefore it is the fastest adopted imaging technology in the history of ophthalmology. This is mainly due to the ease of optical accessibility of the human eye, OCT’s exquisite depth sectioning performance at the micrometer level, and a significantly better performance compared with the previous gold standard in this field, ultrasonography. Furthermore, it is also due to the fact that the human retina cannot be biopsied and finally to the continuous clinically relevant improvements of this technology, due to an exquisite ecosystem between industry and academia in terms of resolution, speed, wide-field imaging, and longer wavelength for choroidal imaging. Motion contrast-based angiography, cellular level retinal visualization, visible light OCT for oximetry and unprecedented retinal layer detection, functional and contrast enhanced extensions, and artificial intelligence (AI)-enhanced performance also contributed to this success. Most of these superb technological developments can be directly translated to the original motivation and idea of OCT: to enable optical biopsy, i.e., the *in situ* imaging of tissue microstructure with a resolution approaching that of histology but without the need for tissue excision and preparation, allowing for quasi-instantaneous diagnostic feedback for physicians, and thereby reducing healthcare costs. There is no doubt that outside ophthalmology, OCT faces significantly bigger challenges with extremely well performing, long-established diagnostic techniques. Hence, OCT has successfully penetrated into different medical fields outside of ophthalmology, but in the last 30 years, it has not been as successful as in ophthalmic diagnosis. Despite the unprecedented success of this imaging technique in ophthalmology so far, there are still numerous remaining challenges in this field to be addressed (e.g., 4D intrasurgical OCT, portable, handheld OCT, and OCT-based digital adaptive optics) but one of the biggest perspectives for OCT is to further push performance frontiers of all involved technologies to converge to the original motivation of OCT, which is to enable *in situ* optical biopsy, especially for early cancer diagnosis and for a better understanding of oncogenesis.

Consequently, this perspective will focus on the following areas that will pave the way for enabling even further enhanced medical diagnosis using OCT in the future. Imaging speed is absolutely essential in medical diagnosis: on the one hand, to minimize the exam time for the patient, but foremost to enable motion artifact free, properly sampled data sets. The speed of today’s systems already supports three- and even four-dimensional imaging as well as wide fields of view and functional extensions of OCT, such as OCT angiography. In the future, different technologies will enable increased OCT imaging speed with one of the fundamental decisions being at which scanning speed single-beam raster scanning will be abandoned and scanning beam parallelization will be used. Further challenges of OCT’s unmatched axial and transverse resolution will also be discussed. Similar to combining different radiology and nuclear medicine imaging technologies in current clinical diagnosis, multimodal optical imaging not only enables the “best of both/all worlds” but also compensates for the deficits of OCT (metabolic, molecular sensitivity, penetration depth, and limited contrast). Multimodal imaging applications combining techniques complementary to OCT will more and more be transferred from significantly improved microscopy setups—acting as fast quasi-histological optical biopsies next to the operating room—to the miniaturized endoscopic level with OCT acting like a global positioning system (GPS) by prescreening the tissue at a wider field of view (FOV) with microscopic resolution. Aside from OCTA, no other functional or contrast enhancing OCT extension has accomplished comparable clinical impact in the last three decades. Some more recently developed ones that might accomplish this challenging task, including quantitative OCTA (especially in neuro-ophthalmology), optical coherence elastography (OCE), dynamic contrast OCT, oximetry using visible light OCT, optophysiology—also referred to optoretinography—and AI-enhanced OCT, will be covered in this perspective. In addition, OCT miniaturization for portable, compact, handheld OCT applications, as well as for home-OCT and self-OCT, will be discussed. Finally, industrial translation of OCT, including medical device regulatory challenges, will be reviewed.

## 2 Key-Technological OCT Performance Specifications: Speed and Resolution

### 2.1 Measurement Speed

The acquisition speed of OCT, typically measured in A-scans per second or voxels per second, has been increasing since its inception. This steady increase in speed has supported the continuous expansion of OCT capabilities and applications. Initially, OCT’s predecessors low-coherence interferometry and optical time domain reflectometry had been used to measure distances along the sample beam or along an optical fiber.^19^^,^^20^ Early time domain OCT systems for ophthalmic diagnostics then recorded B-scans with an A-scan rate of several hundred hertz.^21^^,^^22^ The introduction of Fourier domain OCT (FD-OCT), however, had the biggest impact on speed as well as clinical usability. It increased the A-scan rate to tens of thousands of A-scans per second. With such rates, volume capture scans and high definition scans, i.e., an averaged B-scan calculated from up to 100 B-scans, replaced individual B-scans in ophthalmic diagnostics. More recently, technological improvements of line scan cameras, tunable light sources, digitizers, and data transfer interfaces pushed the speed further to hundreds of kHz and even MHz A-scan rates.^23^^–^^26^ Such speeds now permit the observation of dynamic processes with live volumetric OCT scans, called 4D OCT, in which the fourth dimension is time.^27^

In theory, both FD-OCT implementations, SD-OCT and swept-source OCT (SS-OCT), have similar sensitivity and should thereby reach similar acquisition speeds. However, in practice, SS-OCT has lower losses in the detection and does not suffer from a depth dependent sensitivity roll-off in the case of long coherence length sources such as vertical-cavity surface-emitting lasers (VCSELs) or akinetic swept sources, introduced by the limited modulation transfer function of the spectrometer. At imaging depths of several millimeters, SS-OCT may therefore exhibit >10  dB higher sensitivity than its spectrometer-based counterpart. In consequence, the highest speed confocal point scanning OCT systems so far have been SS-OCT systems. The fastest reported SSOCT systems so far use dispersive stretching of ultrashort pulses propagating through a fiber.^28^^–^^30^ These OCT systems are currently limited by missing fast enough data acquisition boards to sample the stretched ultrashort pulses running at up to 100 MHz. The highest voxel rates are reported for SS OCT systems employing a so-called circular ranging technique, which uses a frequency comb spectrum leading after Fourier transform to degenerate depth ranges sampled in parallel.^31^ The most convincing performance for *in vivo* imaging so far has been demonstrated with systems powered by Fourier domain mode locked lasers,^26^ although vertically VCSEL-based systems are gradually closing in concerning speed and bandwidth performance.^32^

Such high speeds, however, come at the price of decreased sensitivity, at least when imaging light sensitive samples in which the illumination power cannot be arbitrarily increased, such as the human eye. Ophthalmic OCT systems therefore rely on fast safety circuits, which monitor the motion of the scanning beam and the illumination power to quickly shut off the laser in case of a failure. This permits treating the scanned beam as an extended source and thereby higher permissible light exposure than with a stationary beam. However, because it takes time to detect an unintended slowdown of the scanners, the maximum applicable power with confocal point scanning systems will soon be reached.

Another factor limiting the speed of point scanning devices is the requirement for fast scanners. To minimize strain of the scanners and maximize scan speed, sinusoidal or spiral scan patterns may be applied; however, this introduces the need for a resampling step in postprocessing.^33^

**Fig. 1:**
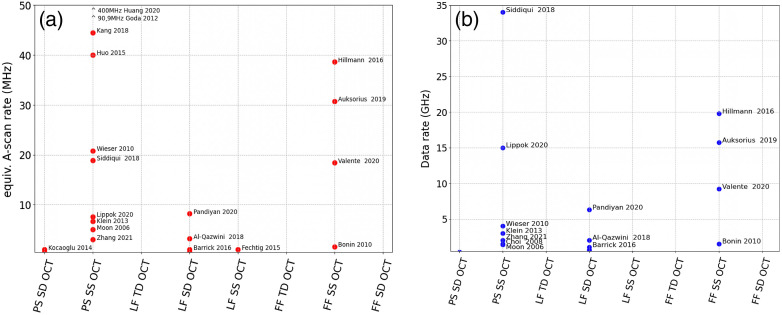
MHz OCT systems: (a) equivalent A-scan rates; for parallel systems, this is the number of parallel channels times the A-scan rate; shown are published systems with rates ≥1  MHz. (b) Data rates in gigasamples/s; only systems with sufficient data available have been included; fastest A-scan rates are achieved by stretched pulse laser OCT; fastest data rates were demonstrated by circular ranging OCT systems. In both categories, full field swept source OCT is a key player (PS, point scanning; TD, time domain; SS, swept source; and SD, spectral domain).

To continue the trend of ever-increasing imaging speed, we see a shift from single-beam confocal point scanning systems toward parallel systems.^34^^–^^36^ These include systems that illuminate the sample with multiple confocal beams in parallel, often referred to as multibeam OCT systems, and systems that illuminate a line or an area on the sample, called line field and full-field OCT (FFOCT) systems, respectively (Fig. 1).

#### 2.1.1 Multibeam OCT

Using a parallel interferometer and multiple confocal illumination spots, one can multiply the acquisition speed of an OCT system by the number of spots. In fiber-optic implementations, this requires multiple interferometers, detectors, and data acquisition channels, as well as a powerful light source.^34^ Although it seems to be a straightforward approach at first glance, the implementation of such systems is not trivial. The coherence gate of the different interferometers ideally should overlap in the sample plane with high precision to image the same depth in all channels. This can be achieved using a variable delay in each interferometer, which however adds significant cost and complexity. In bulk optic interferometers, the multiple beams may share a common interferometer and hence may have inherently matched pathlengths. Implementations based on photonic integrated circuits (PIC) can rely on the high precision of the lithographic manufacturing processes to control pathlength differences. PICs in general are very attractive for multibeam systems because multiplying the interferometer only costs wafer space, which is cheaper than additional fiber interferometers.

From a laser safety perspective, for ophthalmic OCT systems, one can argue that each beam is illuminating a different location on the retina. However, in the anterior segment, the beams are stationary and overlapping. Depending on the combined energy in this “hot spot,” damaging the iris or lens may become a concern.

Another challenge with multibeam systems is merging the acquired multichannel data in postprecessing. In particular, when montaging not only 2D images, i.e, *en face* or B-scans, but also the full volumetric data sets.

#### 2.1.2 Line-field OCT

**Fig. 2:**
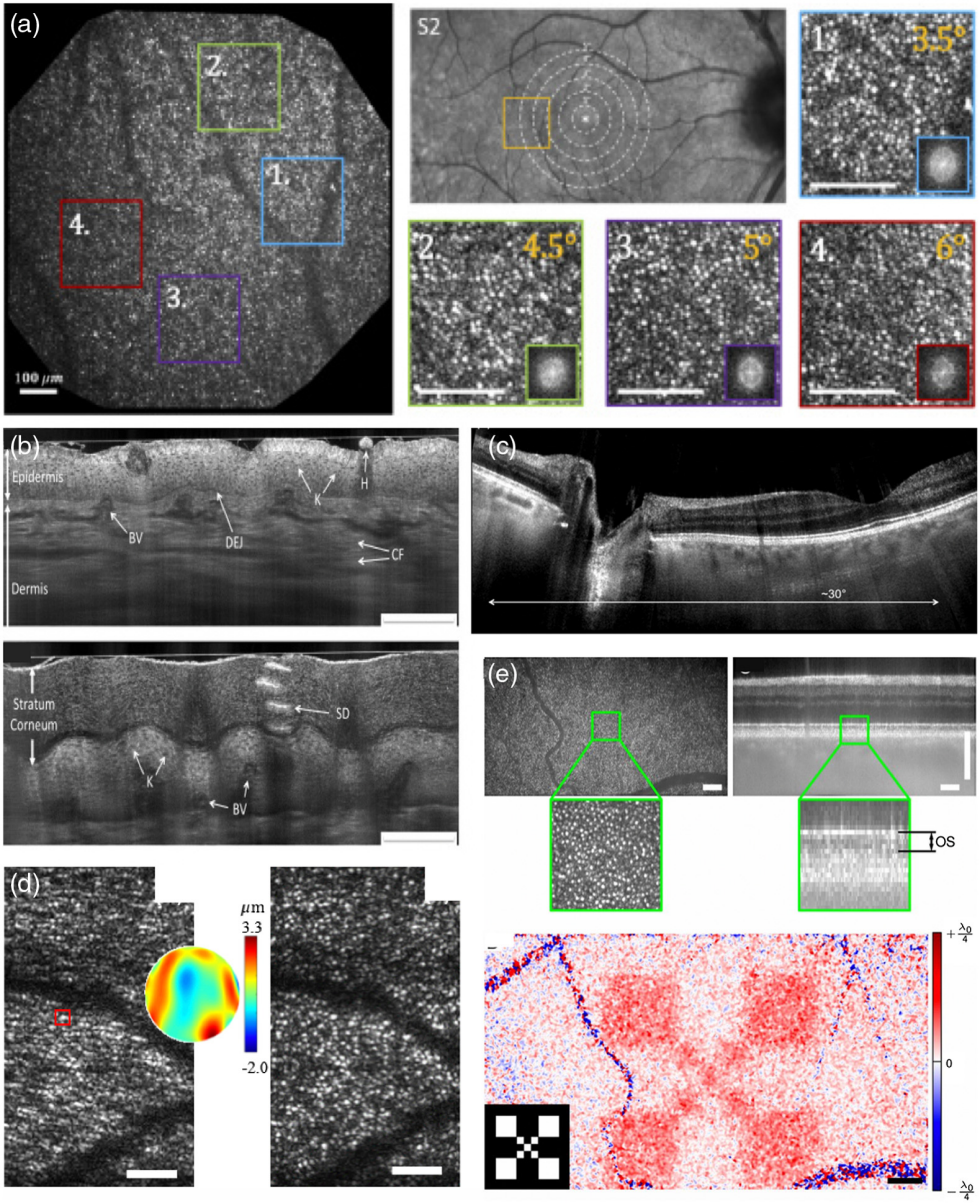
Examples for FF and LF OCT. (a) High-resolution large FOV retinal FFOCT. Panels 1 to 4 visualize the cone photoreceptor mosaic at different eccentricities as shown in panel S2 (scale bar 100  μm). Reproduced from Ref. 42., © 2020 Optical Society of America (OSA). (b) TD LF OCT of human skin, using dynamically aligned confocal and coherence gating (K, keratinocytes; DEJ, dermal–epidermal junction; SD, sweat duct; H, hair; CF, collagen fibers; BV, blood vessels. Scale bars: 200  μm). Reproduced from Ref. 41, © 2018 OSA. (c) Retinal imaging with LF SS OCT at 600 kHz equivalent A-scan rate. Reproduced from Ref. 39, © 2015 OSA. (d) Computational adaptive optics for *in vivo* high-resolution retinal imaging (left: uncorrected enface slice from photoreceptor layer; center: reconstructed wavefront error; and right: corrected enface slice by phase conjugation of the wavefront error to the pupil plane phase; error bars 100  μm). Adapted from Ref. 43. (e) Advantage of FFOCT phase stability for functional OCT assessment of photoreceptor response (upper row: enface plane at OS photoreceptor layer of recorded volume together with tomogram; lower row: response measured as phase change over time after light stimulus onset; inlay shows the actual stimulus mask). Adapted from Ref. 44.

Line-field OCT (LFOCT) comes at all flavors of OCT: TD-OCT, spectral domain OCT (SD-OCT) and swept source OCT (SS-OCT) implementations have been demonstrated.^35^^,^^37^^–^^40^ Its line illumination has a major advantage: because an entire B-scan is captured at once, only one scanner is required to acquire a volume scan. In most cases, this scanner can even have a lower performance than typical galvanometric scanners used in point scanning systems as the scan in the orthogonal direction relative to the B-scan is typically relatively slow. Further, a line illumination maintains confocality in one dimension and never focuses to a spot. This is beneficial for the suppression of multiply scattered photons and permits high illumination powers when imaging the human retina. TD LFOCT particularly allows for keeping the confocal gate aligned with the coherence gate. Using a high-resolution system with tight coherence gating, such confocal LFOCT, has enabled the production of impressive images of human skin that efficiently suppress scattering^41^ [Fig. 2(b)].

In SD-OCT configurations, B-scan rates of several kHz can be achieved by employing standard off-the-shelf 2D cameras.^43^^,^^45^ Such high B-scan rates make LFOCT attractive for functional extensions that require fast repeated scans, such as elastography, OCTA, or Doppler OCT (see Sec. 4). However, high acquisition speed in general is beneficial for achieving high phase stability, making LFOCT also a candidate for phase sensitive extensions, such as computational adaptive optics^43^^,^^46^ [Fig. 2(d)], dynamic OCT, or functional OCT^47^ (see Sec. 4).

SS-OCT implementations require very fast line scan cameras. The fastest suitable off-the-shelf line scan cameras reach line rates of ∼300  kHz. Assuming 1000 spectral sampling points, this results in a B-scan rate of 300 Hz. The number of sampling points can be reduced using an holographic off-axis approach, resulting in full range imaging with up to a 1 kHz B-scan rate^39^ [Fig. 2(c)]. Yet, the lower axial sampling poses limitations on its practical use for clinical imaging. Although the B-scan rate is comparable to state-of-the-art commercial point scanning OCT systems, the low spectral sampling rate may pose problems in the case of fast sample motion. It introduces a phase shift across the spectral interferogram, which results in a point spread function broadening, similar to a dispersion mismatch between reference and sample arm. For example, for retinal line-field imaging, using B-scan rates of several kHz^43^ has been recommended to avoid axial blurring. Recently, a high-speed CMOS camera has been applied for fast line field sensing to overcome the motion limitation, but at increased costs for the sensor.^47^^,^^48^

The advantages of LFOCT and its technical feasibility using readily available components make it the most promising candidate for parallel OCT embodiments and put it closest to market translation.

#### 2.1.3 Full-field OCT

FFOCT permits very simple system designs.^49^ Without the need for scanning devices, a 2D array camera records the backscattered light from the sample as well as from the reference arm. Additional mechanisms however are necessary for extracting the coherently depth gated cross correlation signal between sample and reference light, comprising the OCT signal. TD-FFOCT is equivalent to holography with a broadband source.^50^^,^^51^ The OCT signal retrieval is achieved either by phase-shifting techniques in an in-line configuration or by off-axis using spatial filtering in postprocessing. The in-line approach has the advantage of exploiting the full sampling provided by the 2D array pixels, but comes with the drawback that several phase-shifted image copies are needed for OCT reconstruction. Although the focal plane can be locked to the coherence gate in TD-FFOCT, this advantage is lost in FD-FFOCT. Another advantage of time domain implementation is that it can be cheap, using readily available area cameras.^52^ The advantage of the Fourier domain version on the other hand is its speed,^53^ but it requires high-speed cameras, capable of several hundred kHz frame rate, which are still very expensive.

An important advantage of FFOCT is its intrinsic phase stability across the full *en face* FOV. This makes it especially interesting for phase sensitive extensions of OCT, in which fluctuations in the phase or complex signal are measured over time across repeated *en face* images or volume acquisitions. These include dynamic OCT,^54^ which provides a cell type specific contrast by detecting small oscillations of subcellular components (see Sec. 4.4), functional OCT,^44^ in which changes in the optical length of neural cells in the retina are measured in response to light stimuli [Fig. 2(e)] (see Sec. 4.2), or computational adaptive optics,^55^ in which the phase slope of the wavefront is extracted to measure and correct aberrations (see Sec. 2.2).

In particular, TD-FFOCT implementations often use spatially incoherent light sources that provide intrinsically aberration free imaging over the full FOV^56^ [Fig. 2(a)]. They further suppress cross-talk present in systems using spatially coherent sources, which is beneficial for imaging highly scattering samples, such as brain tissue. To overcome the missing confocal gating mechanism in FD-FFOCT and suppress multiply scattered light, it has been demonstrated to be advantageous to deteriorating the spatial coherence.^36^^,^^57^^,^^58^ This can be achieved using rotating scattering discs, spatial light modulators, or multimode fiber mode scramblers.

Although FFOCT systems typically illuminate a large area on the sample and thereby enable a substantial increase in illumination power when imaging light sensitive tissue, special attention must be paid when the sample is the human retina. To illuminate a large area on the retina, a focus forms in the anterior segment. Critical energy densities are reached in this hot spot soon before the maximum permissible exposure for the retina can be reached. This severely limits the benefit of FFOCT’s large degree of parallelism relative to an LFOCT system when imaging the human retina.

### 2.2 Lateral Resolution in OCT

The lateral resolution of OCT systems is given by the numerical aperture (NA) of the imaging optics. In contrast to, for example, confocal microscopy, it is decoupled from its axial resolution (see Sec. 2.3).^59^ Most FD-OCT systems are therefore designed with a comparably low NA to create a large depth of focus and thereby enable an instantaneous imaging depth of several millimeters.

Diffraction limited resolution can be achieved when the sample itself does not introduce wavefront aberrations. However, especially when imaging the human retina, the imperfect optics of the eye introduces large wavefront errors that prevent diffraction limited resolution when making use of their full NA. It has been shown that the diffraction limited resolution can be recovered by the use of adaptive optics,^60^ using deformable mirrors, or computationally by using the interferometric phase that different approaches have reported, ranging from algorithms from diffraction tomography^61^ to iterative^62^ and non-iterative approaches^63^ to extract and manipulate the wavefront shape. This enables the resolution of individual cells in some retinal layers, i.e., photoreceptors and retinal pigment epithelium (RPE) cells. Although other retinal cells, in theory, are large enough to be resolved even by standard OCT systems, such as ganglion cells, they often remain hidden due to limited contrast. However, it has been shown that averaging a large number of acquisitions or applying dynamic contrast methods helps to reveal them even in the living human eye.^64^

In confocal systems, hardware-based adaptive optics has the advantage of correcting the wavefront optically before the light is coupled back into a single-mode fiber, thereby maximizing the signal. Deformable mirrors, especially ones with a large number of elements, are costly, which so far has prevented their use in commercial OCT systems and makes computational approaches more attractive. To overcome the prohibitive cost of hardware-based adaptive optics OCT on the one hand and the limited collection efficiency of confocal computational adaptive optics OCT systems on the other hand, more basic deformable mirrors may be used to correct low-order aberrations optically and higher orders numerically.

The detection of an aberrated wavefront is not a concern in FFOCT, as long as the detector has sufficient resolution to resolve the wavefront’s phase slope. Because computational wavefront correction algorithms can propagate the focal plane to any depth, they can realize a depth invariant lateral resolution, whereas high NA hardware-based adaptive optics OCT systems are limited to a depth of focus of only a few micrometers.

Cellular and subcellular resolution is the field of optical coherence microscopy (OCM). TD OCT is a natural candidate for OCM since the tight confocal gate can be dynamically aligned with the coherence gate during depth scanning.^65^ For FD OCT configurations with a fixed reference arm, other means are needed to extend the FOV beyond the tight confocal gate. Again, computational methods have shown their merit for OCM by extending the focus depth.^66^^,^^67^ These include hardware-based methods used depth fusion approaches,^68^ Bessel beams,^69^ structured illumination,^70^ or employed metalenses.^71^

### 2.3 Axial Resolution in OCT

The axial resolution of OCT is given by the center wavelength and spectral bandwidth of the illumination.^59^ The most prominent wavelength band of OCT systems lies at 840 nm. This is a result of OCT’s success in ophthalmology. The near-infrared light permits good penetration through the water-like filled eye to the retina, while not blinding the patient and still maintaining good detection efficiency with silicon cameras. Most OCT in this wavelength band are SD-OCT systems using super-luminescence diodes or Titanium:sapphire lasers. With full-width half-maximum bandwidths of up to 180 nm, an axial resolution down to 1  μm in tissue can be achieved.^72^ However, commercial ophthalmic SD-OCT systems typically employ sources with a much narrower spectrum, realizing an axial resolution of ∼5  μm in tissue. If an axial resolution below 1  μm in tissue is desired, super-continuum sources with spectral bandwidths of several hundred nanometers that stretch far into the visible spectrum can be used (see Sec. 4.3).

Most SS-OCT systems operate at longer center wavelengths, 1060 nm for imaging the posterior segment of the human eye, and 1310 and 1550 nm for imaging more strongly scattering samples, such as skin or brain tissue. The achievable resolution at 1060 nm is typically set by the water absorption window, which limits the useful bandwidth to ∼100  nm, resulting in a typical axial resolution of ∼7  μm in tissue. At 1310 and 1550 nm swept-sources with a broader tuning range are available; however, the achievable axial resolution is similar due to the higher central wavelength.

## 3 Multimodal Optical Coherence Tomography

Multimodal imaging or multiplexed/hybrid imaging refers to the assessment of signals of more than one imaging technique. In multimodal imaging, one of the driving motivations is to combine morpho-functional information—enabling the “best of both/all worlds.” This can be accomplished by either acquiring images at different times (asynchronous) and fusing them through digital image manipulation techniques or simultaneously acquiring images (synchronous) and merging them automatically. Main goals of multimodal or multiplexed imaging are to improve early detection and localization of cancer and better understanding of cancerogenesis. Furthermore, multimodal imaging enables examining more than one molecule or molecular and morphologic information at a time, so cellular events may be examined simultaneously or the progression of these events can be followed in real time.

Clinical optical multimodal imaging has so far been successfully applied in ophthalmic diagnosis including color fundus photography, fundus autofluorescence, OCT, *en face* OCT, OCT-angiography, B-scan ultrasonography, fluorescein angiography, and indocyanine green angiography.^73^ In cardiology, morphological features of NIR spectroscopy-detected lipid-rich plaques using OCT and intravascular US are performed in patients undergoing percutaneous coronary intervention for treatment of an acute coronary syndrome.^74^

Multimodal optical imaging in this perspective not only combines OCT with complementary optical imaging methods but also compensates for the deficits of OCT (metabolic, molecular sensitivity, penetration depth, and limited contrast). In this context, OCT will act (in contrast to other microscopy imaging techniques) like a GPS by prescreening the tissue at a wide FOV with microscopic resolution and then other techniques will zoom in at the subcellular or molecular level to enable obtaining morpho-molecular or morpho-metabolic tissue information.

### 3.1 Non-linear Optical Microscopy and Spectroscopy: Subcellular, Biochemical, and Metabolic

Detection of structural information at the cellular level expands the understanding of tissue environment for *in vivo* studies, but the lack of molecular specificity hampers differentiation between pathologic and healthy tissues with comparable scattering or structural properties. Structural alterations in tissues and cells usually take place only after carcinogenic biochemical changes. OCT contrast can be improved by various implementations. However, molecular specificity including metabolic information at the cellular level is still not easily accessible. Therefore, despite the power of OCT, the sensitivity and specificity to detect pathologic tissue are limited.

This weakness limits the ongoing success of this technology. One solution to overcome this limitation and to address current needs in the life sciences and in the clinical practice is to combine OCT with other non-invasive molecular specific modalities. Label-free spectroscopic and microscopic optical imaging technologies such as Raman spectroscopy (RS)^75^ and MPM^76^^,^^77^ that complement OCT^1^ have been established and extensively applied over the past years. These modalities present comparable contrast to standard histopathological methods,^78^ but no single modality can play the role alone.

Indeed, integrated multimodal imaging provides the possibility of fusing morphological information with metabolic-molecular information in a label-free way, complementing basic observation with multiple specific contrast mechanisms to gain a complete picture of disease, but it is still challenging due to different optics and hardware requirements.^79^ MPM has become an essential instrument for biological and medical research with inherent 3D sectioning capability, subcellular resolution, high sensitivity, molecular and metabolic specificity, and deep tissue penetration compared with confocal microscopy, but compared with OCT achievable FOVs, penetration depths and acquisition speeds are restricted. This weakness can be overcome with novel high-speed MPM approaches achieving kilohertz frame rates by implementing pulse-modulated, rapidly wavelength-swept lasers, and inertia-free beam steering via angular dispersion.^80^ In the future, this could help to match the different needs of OCT and MPM, thus facilitating the combination of these complementary techniques in a single co-registered platform.

Usually, the key technologies to add molecular sensitivity to OCT are RS^81^ and coherent Raman spectroscopy. RS allows for full molecular fingerprinting of tissue offers high specificity. Several variants including non-linear vibrational imaging^82^ with different sophisticated laser sources^83^^,^^84^ and single-laser source approaches^85^^–^^87^ have been proposed, but the main deficit is that real-world applications are often missing due to special treatment of the sample—thin slices of transparent samples with high Raman cross sections to be investigated in transmission or reflection mode. Coherent anti-Stokes Raman scattering efficiently provides images with label-free molecular information from DNA, lipids, proteins, and collagen. Recently, stimulated Raman scattering-spectroscopic OCT was introduced to leverage the spatial and spectral multiplexing capabilities of OCT with the molecular specificity and sensitivity of SRS for label-free fast 3D molecular imaging with a single laser on a single platform.^88^ Two-photon excitation fluorescence (TPEF) microscopy^89^^–^^94^ is another powerful OCT add-on that can intrinsically be merged with SHG^91^^,^^95^^–^^97^ or fluorescence lifetime imaging microscopy (FLIM),^98^^–^^100^ showing augmented contrasts with the same FOV and resolution for all modalities. TPEF microscopy can bring unique additional insight into the mechanisms underlying immune system dynamics and function as cellular motility within the native environment *in vivo*. TPEF microscopy and FLIM achieve ultrahigh isotropic subcellular resolution, enhance chromophore contrast via excitation of fluorescent biomolecules, improve sectioning compared with conventional one photon fluorescence, and metabolic information by imaging endogenous metabolites such as nicotinamide adenine dinucleotide and hydrogen/flavin adenine dinucleotide. Pleomorphism (cell nuclei versus cell size ratio) can be detected with subcellular resolution imaging, and grade classification can be performed. Co-registered OCT and TPEF microscopy has the ability to link specific cellular phenotypes and functions as revealed by TPEF to tissue morphology, thus paving the way between basic research knowledge and clinical observations.^101^

**Fig. 3:**
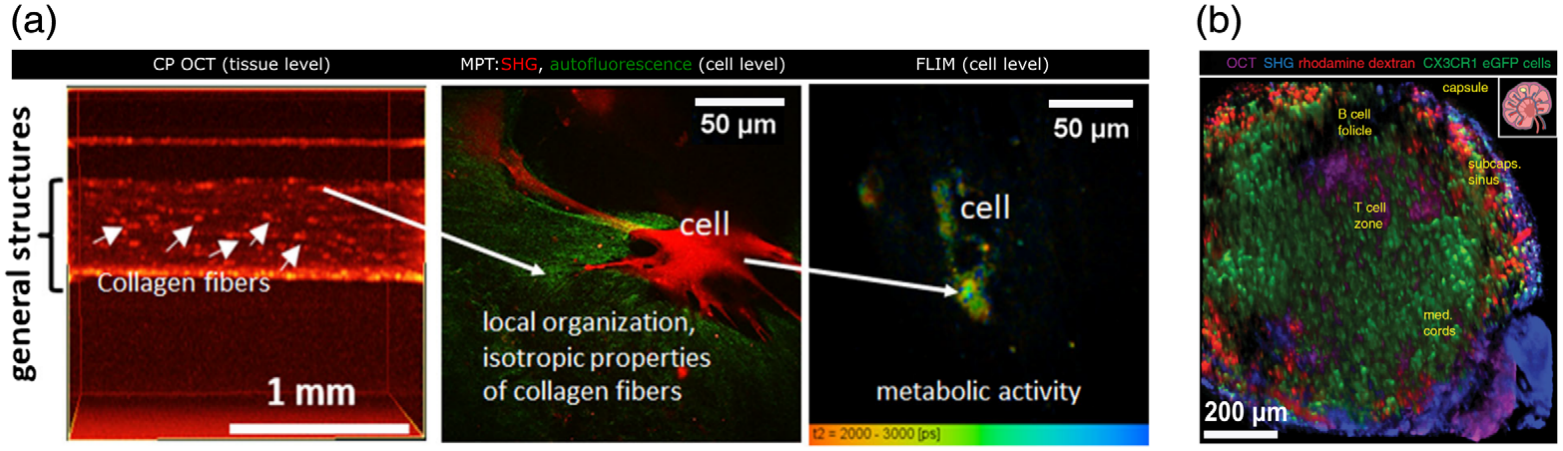
(a) Integrated approach using cross-polarization OCT, multiphoton tomography, and FLIM in skin equivalent preclinical research. Adapted from Ref. 99. (b) 3D reconstructed image in the popliteal lymph node of a CX3CR1:eGFP mouse: OCT (magenta) in the whole lymph node, SHG (blue) of collagen in the lymph node capsule, GFP fluorescence of CX3CR1+ cells (green), and rhodamine dextran signal in blood vessels in the whole lymph node and phagocytes, especially in the subcapsular sinus (red). Adapted from Ref. 101. Scale  200  μm.

SHG provides contrast from non-centrosymmetric molecules such as collagen, which mainly appears in the extracellular matrix as distinct morphological feature. The supramolecular organization can be revealed. *In vivo* skin imaging on a cellular level proves the potential for dermatology.^102^^–^^104^ THG microscopy provides cellular morphological information in real-time with acceptable penetration depth and ultrahigh isotropic subcellular resolution, but reduced penetration depth and remarkable smaller FOV compared with OCT (Fig. 3).

The problems with these multimodal optical imaging approaches with MPM are the need for very high-photon concentration in space and time and subsequent requirement of extremely high near infrared (NIR) laser intensities. Ultra-short laser pulses provide high-peak powers necessary to achieve appropriate excitation powers for MPM signal generation with moderate time-averaged illumination doses. Ultra-broadband Titanium:sapphire lasers, which are proven and well-established light sources for MPM, are a challenge in the commercialization of such combined platform since they introduced high costs and complexity.^105^^–^^109^ Alternative stable and cost-effective light sources are required.^110^ Direct diode-pumping of mode-locked Titanium:sapphire lasers^111^ and scaling up of the achievable output power^112^ could pave the way toward more widespread application of this technology beyond scientific research.^113^ Recently, advancements in soliton-based photonic integrated frequency comb soliton microcombs have accelerated the development of broadband and low-noise chip scale frequency comb sources with the potential for high-resolution OCT deep tissue imaging at 1300 nm.^114^ Combining OCT with MPM at these longer wavelengths or even at 1700 nm can enhance tissue penetration. The key enabling elements to improve the imaging penetration depth, depth of focus, and spatial resolution simultaneously are the laser wavelengths and pulse energy, beam shaping concepts, detection schemes, and real-time AI algorithms. First, deploying longer wavelengths, e.g., 1700 nm for OCT, TPEF, SHG, and THG, the penetration depth for imaging is significantly improved, facilitated by the novel ultrafast laser wavelength agility. Second, novel beam shaping allows for propagating invariant light fields that achieve increased depth, thereby improving penetration and retaining high resolution. Third, to compensate for attenuation intrinsically present in tissue, absorption correction is applied, and, more importantly, dynamic changes from the strong optical scattering are compensated for by so-called computational adaptive optics. The latter relies on OCT motility contrast as a measure of the wavefront for correcting the input and real-time data processing. Penetration depth could be even further improved using light sources at longer wavelengths. Recently mid-infrared OCT could be demonstrated with an axial resolution of 8.6  μm.^115^ Detection could be performed with a standard CCD camera upon implementation of an upconversion module.

One major consideration for future development of the multimodal morpho-molecular metabolic imaging platforms lies in the capability of performing fast OCT and successive simultaneous MPM in the backward propagation direction without affecting one modality to interfere with the others, thus compromising image quality. Hence, one solution in multimodal diagnostic imaging is the combination of a wide-field high-resolution imaging platform including OCT and advanced microspectroscopic imaging techniques and an automated software to extract and classify the morpho-molecular metabolic patterns with the aim to see what can be seen in immunohistochemistry from the macroscopic level to the highly specific microscopic level. With deep learning, automatized full tissue characterization on a multiparametric level can reveal the early onset of disease and improve understanding.

However, deep optical imaging, such as deep brain imaging *in vivo*, at high resolution still poses a great challenge beyond the light source development. Adaptive optics based on direct wavefront sensing can recover diffraction-limited resolution corrupted when achieving deep optical imaging. Successful implementation of adaptive optics in any optical system is always an engineering challenge and often makes the overall system complex and economically expensive. The subaperture correlation-based computational adaptive optics method, which is the computational equivalent of the Shack–Hartmann wavefront sensor (SHWS), can achieve near diffraction-limited performance in FF-SS OCT.^116^ The advantage of this method is that, unlike other optimization-based techniques, it is non-iterative in nature and it does not require *a priori* knowledge of any system parameters such as wavelength, focal length, NA, or detector pixel size. This method can be also extended to region of interest (ROI)-based aberration correction to achieve diffraction-limited lateral resolution beyond the isoplanatic patch in high-resolution point scanning OCT with high NA. A computational sensorless adaptive optics strategy including OCT could play a critical role in correcting aberrations over large volumes and enabling rapid random-access multiplane imaging without highly sophisticated precompensation, SHWS, or electron-multiplying charge-coupled device.

AI-supported denoising and deconvolution of images to increase sharpness, resolution, or brightness are currently implemented at some microscopy platforms. However, it is often limited to a single modality and leaves the user patching different AI systems together for various use scenarios. At the moment, each imaging method needs a specific deep learning architecture with little similarities between each architecture. Moreover, to achieve a good level of validity, one needs a high number of training data to train an AI. One main problem for achieving the needed quality for detection and pixel-based quantification through deep learning lies in the lack of quality in ground truth data and the lack of a ground truth management system. By invoking a novel ground truth management system, an efficient AI system can be trained using only a few training data sets. Once the AI has been trained for one modality, the gained “experience” can be used to significantly speed up the training for other modalities through new transfer learning techniques, i.e., passing on experience. Active learning and knowledge transfer can improve learning speed and accuracy. This will apply to resolution enhancement as well as automatic annotations for ROIs. Therefore, in a unified approach in which the AI system supports OCT and MPM, radically improved learning speed and accuracy can be provided to establish novel multiparametric biomarkers. By developing and deploying novel beam shaping concepts, supported by advanced AI algorithms in combination with real-time data processing, critical parameters for the imaging performance in terms of penetration depth, acquisition speed, and spatial resolution are vastly improved, which could pave the way toward a compact multimodal biophotonics platform for advanced real-time 3D intrasurgical morphological, metabolic, and molecular imaging platform with increased sensitivity and specificity.

### 3.2 Photoacoustic Microscopy and Tomography: Absorption, Molecular Contrast, and Seeing Deeper

OCT as an optical imaging modality that relies on the optical scattering properties of samples has its inherent limitations in terms of contrast generation. The strong scattering of many biological tissues further restricts the penetration depth of OCT due to its dependence on ballistic photon detection. To alleviate these problems, combining OCT with PAI has been explored by various groups over the past decade. Our previous review paper covered this topic for scientific works up to 2014.^117^ This perspective focuses on an update on dual modality OCT/PAI system configurations published recently as well as an outlook focusing on their application in molecular imaging.

#### 3.2.1 System configurations for dual modality OCT-PAI

Combining OCT and PAI can be categorized into three major implementation schemes: OCT with photoacoustic microscopy (OC-PAM), OCT with photoacoustic tomography (OC-PAT), and OCT with photoacoustic endoscopy (OC-PAE). Each of these schemes is briefly discussed below.

In terms of OC-PAM, ever since the first realization of this technique,^118^ piezoelectric transducers have been the go-to solution for photoacoustic signal detection. Over the past few years, we can still see the piezoelectric transducers being used in OC-PAM configurations. Among these piezoelectric transducers, needle transducers are most commonly used. So far, OC-PAM using needle transducers has been applied in choroidal and retinal imaging^119^^–^^127^ extensively. Some technical advancements of needle-transducer-based OC-PAM systems have also been reported such as for dynamic focusing^128^^,^^129^ and for incorporating additional imaging modalities.^130^ However, due to the opaque nature of the needle transducer and knowing that these needle transducers are normally unfocused, alternatives for OC-PAM implementation have been explored. One direct approach is to make the transducer transparent, which was recently demonstrated in an OC-PAM system and applied in different disease or pathological models.^131^ Another approach would be to use optical detection for the photoacoustic waves. Using Michelson interferometry, OC-PAM was achieved and characterized.^132^ Using the principle of Fabry–Perot interferometry, an akinetic photoacoustic sensor was demonstrated^133^ and henceforth applied in OC-PAM imaging.^134^^,^^135^

For OC-PAT, the implementation is still based on a system using a polymer film sensor.^136^ After the successful application of this system in chick embryo imaging^137^ and human skin imaging,^138^ the functional extension of OCTA was added^139^ and demonstrated in clinical settings.^140^^–^^142^ The current development is to increase the speed of acquisition to match the two modalities’ acquisition time.^143^ Other photoacoustic pulse sensing methods, such as a microring resonator, were also explored to be incorporated into OC-PAT systems.^144^ As for OC-PAE, after the first published configuration using a transducer,^145^ an improved version of the probe was introduced as a proof-of-concept design.^146^ Using all optical detection, an OC-PAE probe for intravascular imaging was demonstrated.^147^ A more detailed review on dual-modality imaging using OCT and PAI was recently published.^148^

#### 3.2.2 Contrast agents in OCT and PAI

Endogenous contrast is not sufficient for fully exploiting PAI’s great potential for the visualization of physiology and pathology at the molecular level. Therefore, the development of imaging probes became an utterly important field for research. The use of specific multimodal OCT-PAI probes is often not necessary since PAI’s molecular imaging capabilities nicely complement OCT’s superiority in morphological imaging.

Signal compounds for PAI can be divided into three broad classes: small-molecule dyes, inorganic, and organic nanostructures.^149^ Organic nanostructures and especially semiconducting polymer nanoparticles gained elevated interest since they can be easily functionalized and are of relatively low cost and potentially biosafe.^150^^,^^151^ In addition, they possess excellent photostability and a high mass absorption coefficient.^152^

PAI probes can be used to image deeper into tissue to, e.g., allow for whole body mouse, rat, and human organ imaging,^153^^,^^154^^,^^154^^–^^160^ to enhance the signal-to-background ratio,^161^ for phototherapy and photoactivation,^151^^,^^162^ and for molecularly targeted imaging.^149^^,^^152^^,^^158^

Molecular targeting can involve the qualitative or quantitative detection of potential biomarkers such as reactive oxygen species,^163^^,^^164^ pH,^165^^,^^166^ ${\mathrm{Ca}}^{2+}$,^167^^–^^171^ matrix metalloproteinases,^172^ and granzyme B.^173^ Biomarker imaging has mainly been demonstrated in animal models for various types of cancer, sentinel lymph nodes, liver dysfunction, and PAI of T lymphocytes, whereas intraoperative multimodal pancreatic cancer detection was already performed in humans.^174^

The combination of OCT with molecular PAI is still in its infancy, but it can offer new insights into pathophysiological processes. Targeting drug tolerant persister cells with signaling compounds in animal models and organoids might be a promising application for a combination of PAI and OCT for both tomography and ultrahigh-resolution imaging to aid cancer therapy.^134^^,^^144^^,^^175^^,^^176^

Molecular PAI can be exploited to investigate a recently discovered fluid drainage pathway in the eye.^177^^,^^178^ Monitoring of the ocular drainage rate into the lymphatic system can potentially be used as a measure for the effectiveness of existing and novel glaucoma treatments,^179^^,^^180^ and a combination with OCT and retinal blood flow measurements could promote our understanding of glaucomatous optic neuropathy.

#### 3.2.3 Outlook of OCT-PAI

**Fig. 4:**
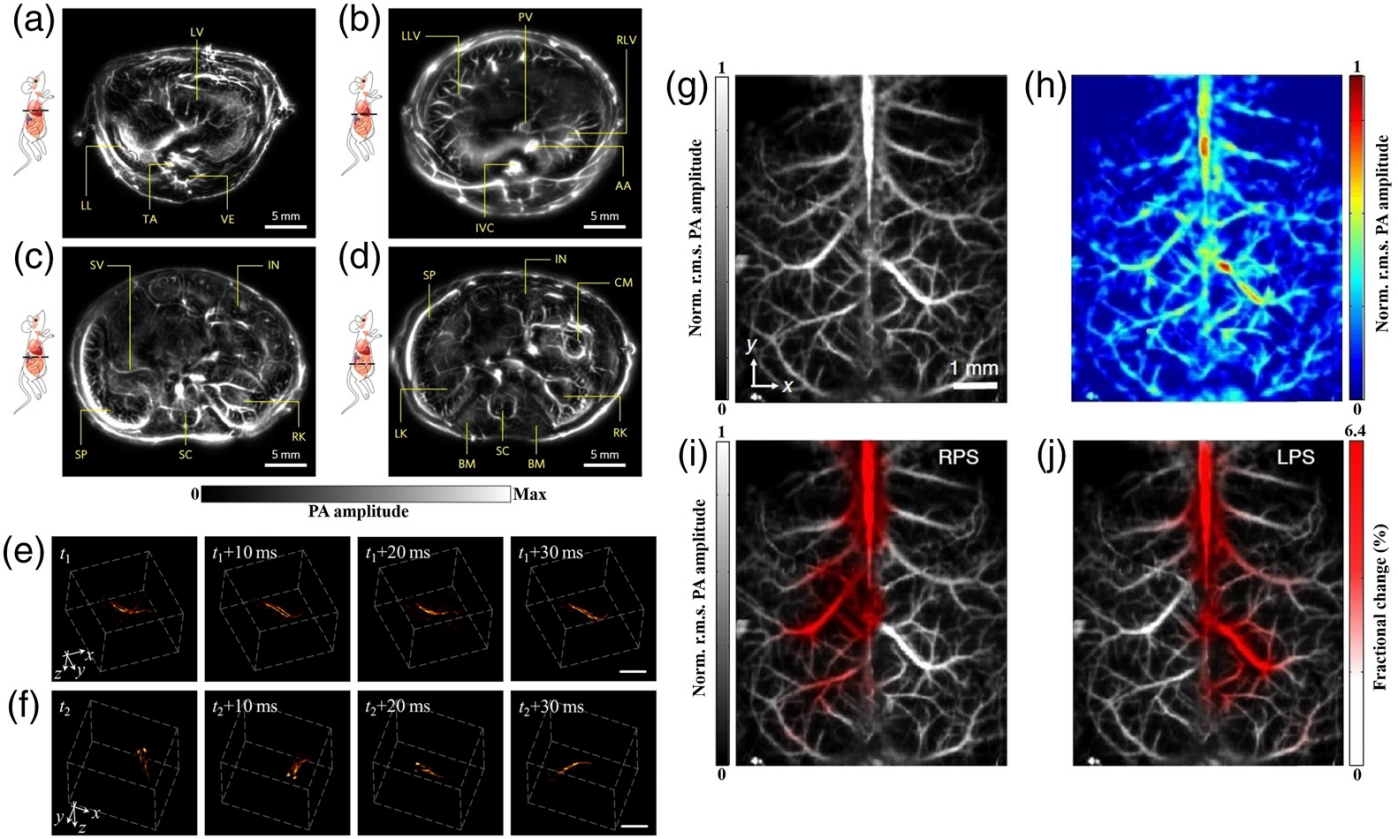
Real time PAI: (a) cross-sectional images of the lower thoracic cavity, (b) two lobes of the liver, (c) upper abdominal cavity, and (d) lower abdominal cavity of a nude mouse at a frame rate of 50 Hz. AA, abdominal aorta; BM, backbone muscles; CM, caecum; IN, intestines; IVC, inferior vena cava; LK, left kidney; LL, left lung; LLV, left lobe of liver; LV, liver; PV, portal vein; RK, right kidney; RLV, right lobe of liver; SC, spinal cord; SP, spleen; SV, splenic vein; TA, thoracic aorta; and VE, vertebra. Reproduced from Ref. 154 with permission from Springer Nature. Time-lapse 3D images of a freely swimming zebrafish recorded at an imaging rate of 100  volumes/s. The shown sequences correspond to two portions of the movie having (e) smooth and (f) abrupt movements. Scale bar is 1 mm. Adapted from Ref. 188 with permission. Wide-field imaging of mouse brain hemoglobin responses to front paw stimulations at 2 kHz frame rate using a single transducer and an ergodic acoustic relay. (g) Calibration image of mouse brain vasculature through an intact skull. (h) Wide-field image. Fractional changes in signal amplitude (shown in red) in response to (i) right paw stimulation and (j) left paw stimulation superimposed on the calibration image. Norm., normalized and r.m.s., root mean square. Adapted from Ref. 186 with permission from Springer Nature.

A major technological limitation of current OCT-PAI systems is the imaging speed mismatch between the two modalities.^1^^,^^148^ Although modern OCT technology permits video rate imaging, PAI systems, especially when PAT is applied, are much slower. Recent advances in high-speed PAI have pointed the way for novel real-time OCT-PAT systems. First, as the speed of PAI has been largely confined by the limited repetition rate of current pulsed laser sources, high repetition rate light sources such as pulsed laser diodes^181^ and light emitting diodes^182^^,^^183^ have been applied for *in vivo* PAI. However, the low fluence from these alternative sources has resulted in imaging results with a poor contrast, which could be potentially improved by emerging methods such as deep learning^184^^,^^185^ (Fig. 4).

Another way toward real-time OCT-PAI systems is applying multiple transducers and parallel data acquisition for PAI, which has demonstrated 2D PAI for large objects^154^^,^^187^ and 3D PAI for a smaller FOV.^188^ However, currently most photoacoustic detectors are conventional piezoelectric transducers, which are opaque to OCT detection beams. Therefore, all optical US detectors, due to their optical transparency, are especially interesting for OCT-PAI systems. Using a planar Fabry–Perot etalon, a multibeam scanner for simultaneous interrogation of multiple points was demonstrated.^189^ This parallel detection reduces the 3D PAI time to a few seconds. Increasing photoacoustic interrogation beams will further increase imaging speed.

However, the parallel detection also greatly increases system costs. A new trend in high-speed PAI is to reduce acoustic detection points and adopt novel algorithms for image reconstruction using the obtained sparse data. These methods can be classified as iterative reconstruction algorithms^190^^–^^193^ and deep learning methods.^194^ For the former, prior knowledge of photoacoustic images, such as smoothness, sparsity, or total variation constraints, are added in the iterative algorithms. For the latter, *a priori* training is necessary to reconstruct high-quality images from sparse data.

Furthermore, in the latest breakthrough to speed up PAI, the number of required transducers for a large area 3D imaging was reduced to one single detector.^186^ To do this, an acoustic relay cavity is placed between the imaged object and the detector. The propagation of photoacoustic waves in this cavity creates unique stretched acoustic pulse signatures for each point in the FOV. Therefore, upon full field illumination, the signal of the single detector is the combination of stretched pulses from the whole FOV; thus the optical absorption at each point can be unmixed based on the acoustic pulse signature. Although more research is needed for this method, together with all progress in achieving high-speed PAI, these breakthroughs have paved the way to future real-time OCT-PAI technologies.

### 3.3 Multimodal Endoscopic OCT: More Comprehensive Access to Internal Body Organs

In addition to its success story in ophthalmology, OCT can also provide exquisite cross-sectional morphological information of organs that are not easily accessible, such as coronary arteries, intestines, or the brain. OCT imaging penetration suffers from light attenuation in tissue, especially due to high scattering at the near-infrared wavelengths (800 to 1300 nm). Penetration depths of ∼0.5 to 2 mm prevent OCT from acting as a full-body imaging modality but enable tissue information up to comparable depths of those accomplished with conventional biopsies. In the past 30 years, research and industry have focused on the development of optical probes to endoscopically access internal organs with OCT. Two major approaches have been realized: sideward and forward imaging devices.^195^ Luminal organs, such as vessels, airways, or the esophagus, can be imaged by sideward viewing probes, realized with scanning mechanisms based on micromotor-based distal rotation of a reflector^196^ or proximal scanning of rotary joints.^197^ Larger hollow organs, such as the urinary bladder, stomach, or cervix, are accessible via a forward viewing probe placed in front of the ROI. Beam scanning is achieved using microelectromechanical systems (MEMS), such as piezoelectric actuators^198^^,^^199^ or mirrors.^200^^,^^201^ Also other forward scanning schemes have been reported on paired GRIN lenses^202^ or optical fiber bundles,^203^ although fiber bundles have not yet shown sufficient OCT performance and reduce probe bending flexibility for proper clinical applications.

During the development process of such probes, general optical and mechanical requirements have to be met: overall mechanical diameter, taking safety measures into account for electrical isolation, electromagnetic shielding, bending protection, and sealing; the necessary rigid distal length of the probe to fit clinical instrumentation, which has often insertion angles on the proximal end; or a careful micro-optical design to reach best optical performance. For the lateral resolution, approaches have been proposed to access development parameters already in the biomarker identification phase using microscope setups.^204^ Additional obligatory clinical requirements regarding sterilizability or biocompatibility must be taken into account. Furthermore, orientation is key for performing biopsies at a location identified with endoscopic OCT for proofing diagnosis or resecting identified malignant lesions. A proposed approach in esophageal endoscopic OCT is using laser landmarks.^205^^,^^206^

**Fig. 5:**
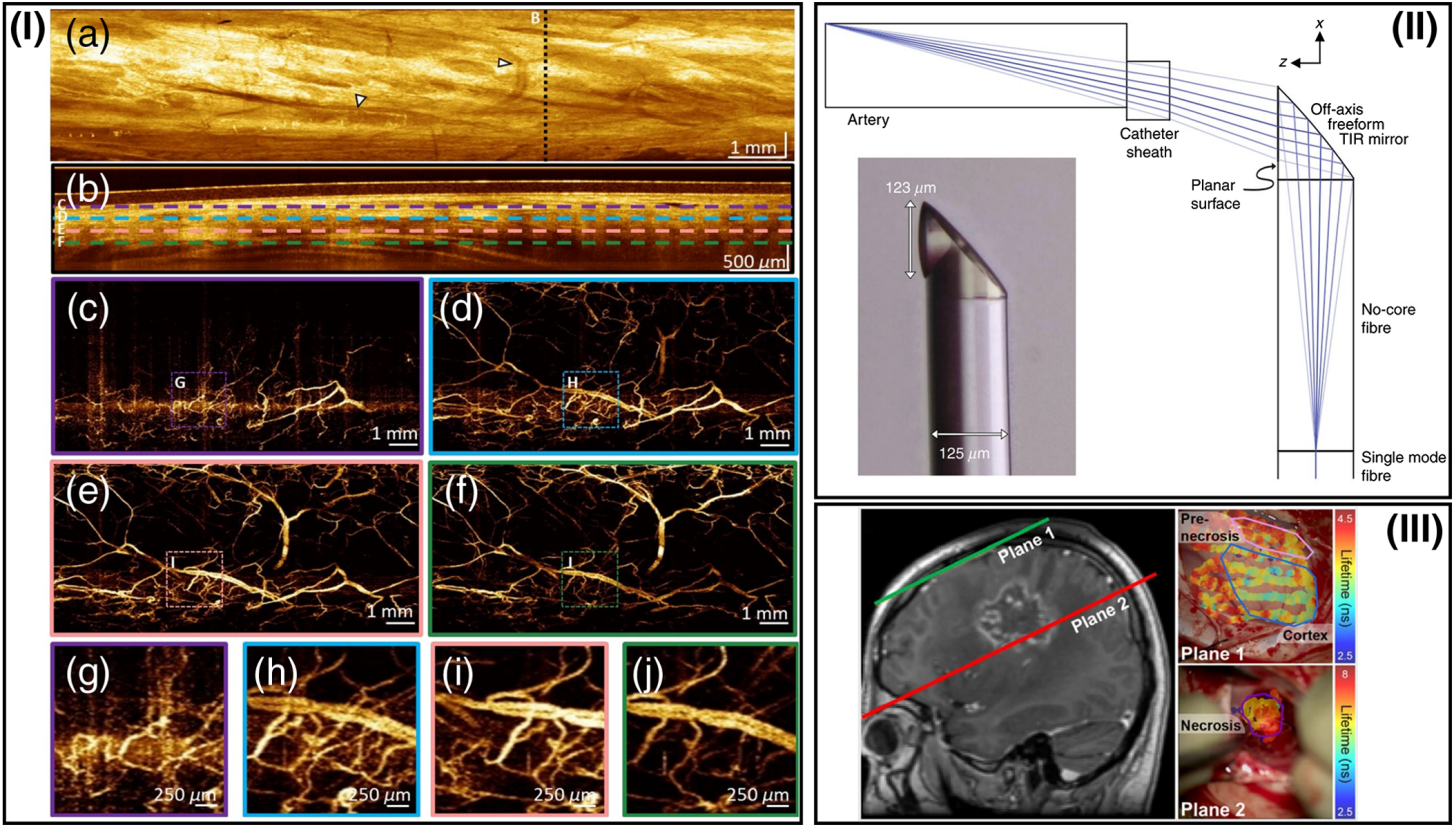
Representative steps toward the development of fast data acquisition, 3D printed optical free-forms and representation easy to follow in clinical routine processes. (I) Zhang et al.^32^ reported on sideward imaging e-OCT and OCTA of a swine esophagus at an A-scan rate of 2.4 MHz. The distance covered by pullback was 14 mm, and a B-scan rate of 600 Hz was achieved. Arrows in (a) indicate blood vessels in OCT enface image, dashed lines in (b) indicate the depth of (c)–(f). This remarkable performance of OCT and OCTA at MHz speed is paving the way toward multi-megahertz endoscopically acquired data of OCT and OCTA. Adapted from Ref. 32. (II) Li et al.^208^ have realized 3D-printed free-form optic directly spliced on a light delivering fiber for improving optical performance (TIR, total internal reflection). Not only are very thin optical assemblies possible with this technique, but also the combination of various complementary techniques might be enabled with tailored optical properties to achieve outstanding imaging quality. Adapted from Ref. 208. (III) Alfonso-Garcia et al.^209^ introduced an augmented visualization of FLIM delineating brain tissue from prenecrotic and necrotic tissue. Left: Magnetic resonance image with indications of planes 1 and 2 of the FLIM maps on the right. Extrapolating this approach toward information feedback to clinicians, the color-coded maps, based on one single or multiple imaging techniques, could be easily interpreted and used by clinicians for immediate medical intervention. Adapted from Ref. 209.

Not only is the change toward a combined diagnostic and therapeutic tool of high interest, but also new approaches in probe design and development have been fostered recently. Concepts based on diffractive lenses were reported by Xi et al.^207^ Although the construction size is still considerably large, diffractive lenses are overcoming the compromised optical performance if OCT is implemented in endoscopic probes. Improved image performance was demonstrated by Pahlevaninezhad et al. in 2018 using metalenses for developing nano-optic endoscopes. These specially designed metalenses were shown to precisely control the light phase, thus reducing spherical aberrations and astigmatism. Therefore, increased depth of focus in parallel to high resolving power is achieved. This technique could furthermore be beneficial for other endoscopic imaging modalities.^71^ As 3D printing currently is available for metal and glass material, the development and research are going toward 3D printed glass surfaces with optical quality. This enables freeform optics manufacturing of optical components and even multilens objectives.^15^ Implementing this technology in optical endoscopic probes allows for direct printing on optical fiber facets with freeform optical elements, such as a freeform total internal reflection mirror for sideward imaging endoscopic OCT.^208^ In addition, microstructuring concepts will most likely lead to implemented anti-reflection behavior of the optical elements and tailored optical properties using different photoresists to customize optical properties at a micrometer scale (Fig. 5).

Endoscopic OCT on its own has great potential in providing cross-sectional morphological information. Nevertheless, it lacks molecular or metabolic tissue contrast. Intramodal multimodal imaging using Doppler OCT, polarization sensitive OCT (PS-OCT), OCE, or spectroscopic OCT may provide additional contrast, but its clinical impact is still unclear.^210^ As an additional extension, endoscopic OCTA for perfusion and angiogenetic contrast is of increasing interest as well. First realized in sideward viewing probes,^211^ forward endoscopic OCTA has recently been successfully applied,^212^^,^^213^ demonstrating promising advantages compared with narrow band imaging with respect to 3D visualization and increased depth.^214^ Increasing clinical endoscopic OCTA usability will require even further stabilization, robust acquisition modes, and image co-registration.^215^^–^^217^ Imaging speed is certainly an important factor for its easy adaption. MHz OCT was reported for intravascular imaging to overcome heartbeat artifacts during imaging.^218^ Multi-MHz OCT/OCTA was reported recently and enables video rate OCT/OCTA at impressive imaging performance.^32^^,^^218^ In the near future, multimodal endoscopic OCT will demonstrate significant potential to unleash the full capability of accessing complementary morpho-functional and/or morpho-molecular tissue information needed for improved clinical diagnosis and therapy monitoring. For example, reports on a probe combining OCT and fluorescence imaging^219^ providing additional molecular contrast and on a probe combining OCT, PAI, and US^220^ have been published. There are still challenges to be solved for a multimodal endoscopic OCT combination: the optic design needs to combine and find the best solution to fulfill the various optical requirements coming from the different image technologies in the scope of limited physical space within an endoscope. The acquisition times for the different modalities differ and are limited by biological constraints such as peristaltic movement, heartbeat, or breathing. Co-registered information is critical to retrieving the correct clinical information, especially if FOVs of the combined techniques are different. Research has been conducted toward the full cross-link of OCT and RS, despite remaining challenges.^221^ To investigate clinical validity, large clinical studies, preferably multicentral studies, are absolutely necessary. Toward real-time displaying of the relevant information, the complementary techniques should be processed and visualized in a way to permit *in situ* clinical understanding and finally diagnostic decision. Therefore, each single modality needs to be optimized for detecting/characterizing the disease.^222^ Data analysis speed and robustness have to be optimized, enabled by the full capacity of classification algorithms dealing with multivariant analysis. In the near future, computer science involvement will be the major topic for bringing endoscopic OCT (with or without other imaging technologies) to the patient bedside and into daily clinical practice. Techniques such as unsupervised classification for biomarker identification may be promising approaches. Morpho-molecular augmented painting, as presented by Alfonso-Garcia et al.,^209^ with autofluorescence lifetime imaging during neurosurgery, would be an intuitive real-time information display for guiding clinicians to detect the malignant tissue. Finally, this technique would lead to an augmented map for resecting malignant tissue inside human organs based on morphological, molecular, and functional contrast.

From a clinical point of view, the use of the described imaging devices—capable of multimodal endoscopic OCT—should preferably be applicable in an outpatient department setting, where typically no stationary stay of the patient is required, no general anesthesia is necessary, and immediate diagnostic information is needed. To achieve this, further miniaturization and increased usability with minimum patient discomfort are key. A quantum leap in diagnostic imaging of the gastrointestinal (GI) tract, for instance, may be swallowable low-cost imaging units transmitting reports of the GI tract to the patient’s cell phone or even to a centralized data analysis facility. Further development may go toward personalized medicine available at home to everybody for various internal organ diagnostics.

## 4 Extensions of Optical Coherence Tomography

Alike in other (especially microscopic) imaging technologies, numerous functional and contrast enhancing OCT extensions have been developed in the last 30 years immediately after its invention. In the case of OCT, these additional functional and contrast enhanced tissue information come with OCT’s exquisite micrometer depth axial resolution as opposed to an integration over the entire depth penetration. In academia, several successful functional and contrast enhancing OCT extensions have been initiated, but aside from OCTA, a label-free motion-contrast-based functional OCT extension providing perfusion and hence angiogenetic information, no other functional or contrast enhancing OCT extension has accomplished comparable clinical and industrial impacts. One of the first OCT extensions enhancing tissue contrast by collecting light resolved by polarization and thus revealing tissue birefringence is PS-OCT. In 1992, Hee et al.^223^ demonstrated a polarization-sensitive low-coherence reflectometer and characterized the birefringence of a wave plate and *ex vivo* calf coronary artery tissue. More than 500 publications in this field^6^ by numerous academic groups demonstrated the great potential of this OCT extension especially in the eye and the skin,^1^^,^^224^^–^^227^ but so far it has not been successfully translated to a commercial system or proven its diagnostic impact. Catheter, endoscopy, and needle-based PS-OCT might, in the near future, turn out to be an extremely interesting clinical application for this contrast enhancing OCT extension.^228^^–^^230^

Before OCTA, Doppler optical coherence tomography (DOCT) was the first^231^^–^^233^ and most extensively used functional OCT extension determining the speed of moving particles in the tissue by measuring the frequency shift imparted on light scattered by the particles and already setting out to produce three-dimensional maps of tissue perfusion.^1^^,^^234^ The classic example of Doppler shifts is the increase in frequency of an approaching train whistle followed by the decrease in frequency as it passes and departs. Higher sensitivity was achieved by phase sensitive Doppler OCT,^235^ which in combination with higher speed of FD OCT ultimately allowed for measurement of blood flow in a large range of retinal vessels with high sensitivity.^236^ As with other laser Doppler flow measurement techniques, DOCT has several challenges, the most critical being that the accurate measurement of velocity requires knowledge of the angle between the OCT beam and the direction of the velocity in the sample. The first paper introducing the notion of optical coherence angiography applied known methods of DOCT to produce retinal angiographic maps.^237^ Later work established the intuitive notion of OCTA for perfusion mapping. With the persistent split of pure structural angiographic mapping as OCTA from the originally overarching notion of DOCT, DOCT’s scientific output (more than 4000 for OCTA and about 450 for DOCT^6^) and hence commercialization [Thorlabs (Newton, New Jersey) and Optovue, Inc. (Fremont, California)] was consequently reduced.

### 4.1 Optical Coherence Angiography

**Fig. 6:**
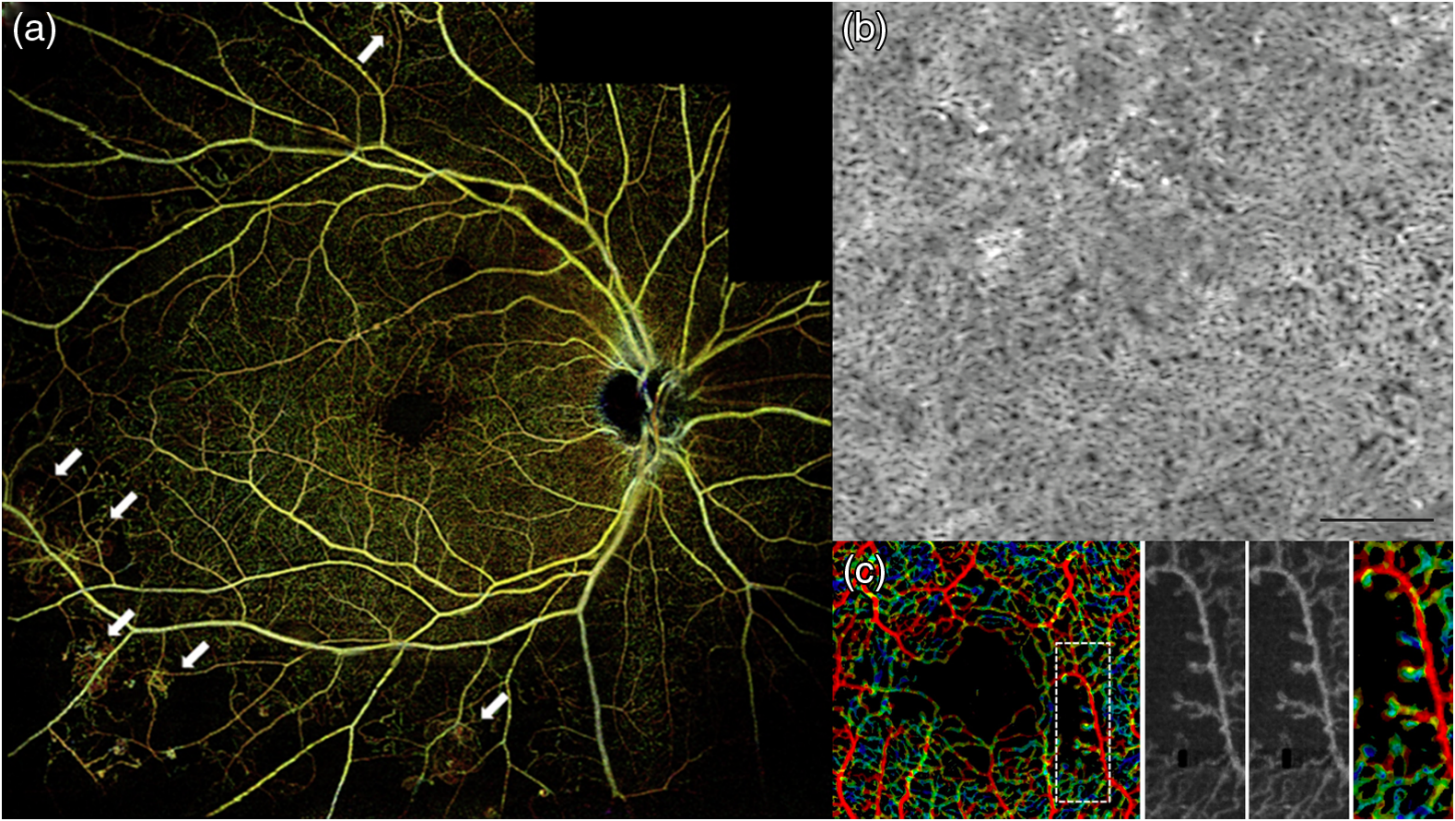
(a) Wide-field OCTA of the human retina: posterior pole montage of 12×12  mm (about 40 deg) swept source OCTA. Adapted from Ref. 252. (b) Averaged OCTA image of the choriocapillaris. Adapted from Ref. 253. (c) OCTA VISTA image: red indicates faster blood flow speeds and blue indicates slower speeds. Enlargements to the right, with two different mean projection times of 1.5 and 3 ms as well as the OCTA VISTA version, respectively. Notice that the capillary loops, which likely correspond to microaneurysms, are associated with slower blood flow speeds. Adapted from Ref. 254.

OCTA is a label-free non-invasive OCT extension that uses blood cell motion contrast for high-resolution imaging of volumetric blood flow information generating angiographic images—hence providing both structural and functional (i.e., blood flow/perfusion) tissue information. Such angiographic maps in 3D have already been demonstrated by FD OCT-based Doppler OCT by several groups.^234^ However, the visibility of small capillary vessels was critically improved by comparing signals of adjacent B-scans rather than A-scans.^238^ Instead of quantifying the correlation between signals, OCTA compares the decorrelation signal between sequential OCT B-scans taken at the same cross-sectional location to construct a map of blood flow. Emerging from Doppler OCT, between 2004 and 2012, at least 10 different research groups published different versions of OCTA, the majority of them claiming its invention and producing new acronyms for OCTA.^237^^,^^239^^,^^240^ The majority of the 4000 publications are in the field of ophthalmic diagnosis.^241^^–^^243^ However, OCTA has also been successfully demonstrated for detecting angiogenetic biomarkers in cancer diagnosis and therapy monitoring as well as in endoscopic applications.^244^^,^^245^ The success in ophthalmic applications and in clinical translation of this technique lies in its technological simplicity, moderate additional engineering as compared with conventional OCT systems, and extremely significant clinical impact—slowly replacing fluorescein angiography and indocyanine green angiography in clinical routine. To eliminate patient or organ movement induced artifacts, OCTA requires higher imaging speeds than most currently available OCT systems. It is noteworthy that OCTA provides 3D qualitative flow information at a fixed point in time. Therefore, vessel leakage is not detectable by OCTA. Furthermore, exact automated segmentation of all diagnostically important intraretinal layers is of essence to avoid artifacts in the OCT angiograms of the respective layers. Consequently, exact segmentation necessitates sufficient system sensitivity, axial resolution, and contrast. Retinal blood flow on OCTA can be obscured by hemorrhage as this decreases the ability of light to penetrate into the deeper layers of the eye. Despite the rapid, tremendous commercial and clinical success of OCTA, some (at least relative) blood flow quantification will be needed in the near future.^246^^–^^249^ Improved and reproducible quantitative OCTA is definitely also of significant clinical interest as is the correct visualization and quantification of choriocapillaris^247^^,^^250^^,^^251^ (Fig. 6).

Another important future clinical role enabled by ultrahigh speed swept sources will be wide-field OCTA for the detection of neovascularization of the disc and elsewhere, microaneurysms, changes of the foveal avascular zone, intraretinal microvascular abnormalities, and capillary non-perfusion.^255^^,^^256^ This advancement of OCTA technology in clinical research will ultimately lead to enhancement of individualized management of diabetic retinopathy and prevention of visual impairment in patients with diabetes.

Using the eye and especially the retina as a part of the central nervous system diagnostically as a window to the brain started in the late 1970s, and about 400 papers since then have covered diagnostic methods in the posterior pole of the eye for early diagnosis of brain diseases. An important prerequisite for successful OCTA-based diagnosis in neurodegenerative diseases and other clinical applications will be accurate and reproducible quantitative OCTA. Quantitative analysis of OCTA is essential to standardize objective interpretations of clinical outcomes.^257^ Indeed, a concerted effort has been put forth to understand how Alzheimer’s disease (AD) pathology may manifest in the retina as a means to assess the state of the AD brain.^258^^–^^260^ OCTA has also been successfully evaluated as a tool to assess retinal changes in Parkinson’s disease^261^ and both schizophrenia and bipolar disorder.^262^

### 4.2 Optophysiology/Optoretinography: Non-Invasive Detection of Intrinsic Optical Signals

**Fig. 7:**
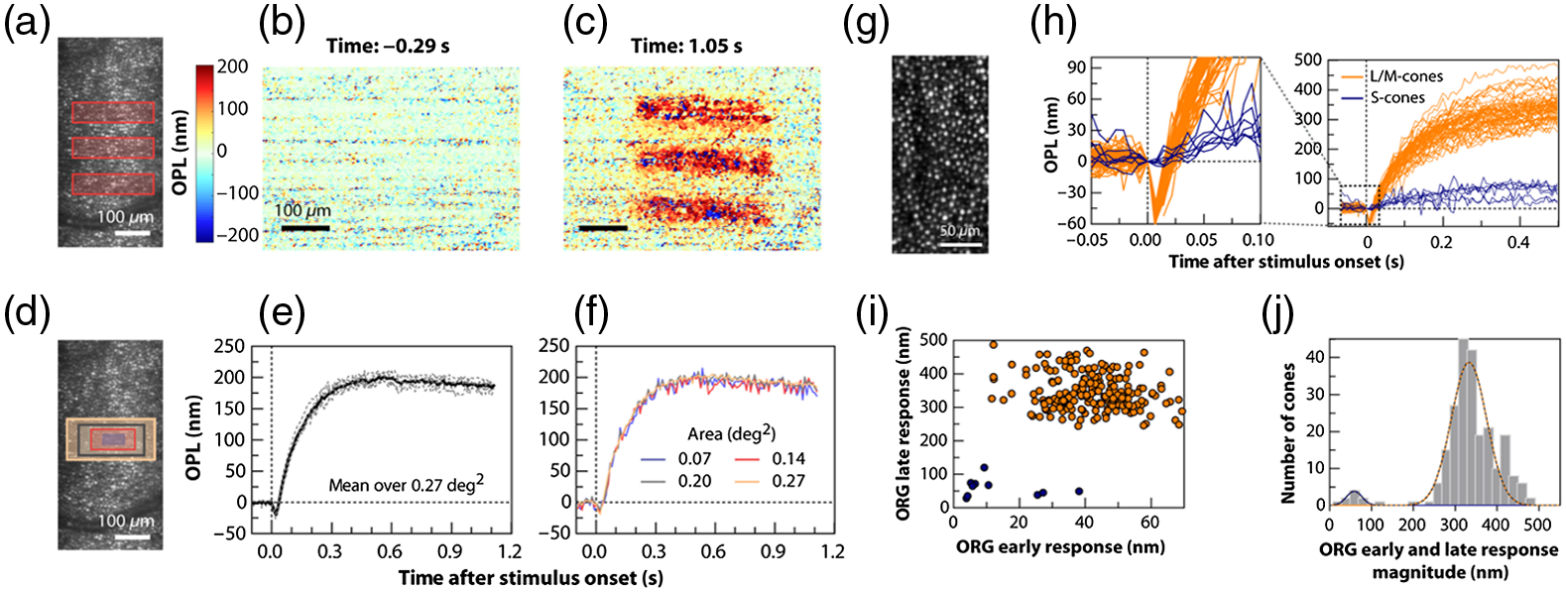
(a) Illumination pattern (three bars) drawn to scale over the LSO image. The spatial map of OPL change between the ISOS and COST (b) before and (c) after stimulus measured at 20 Hz volume rate. (d) Rectangles over an LSO image represent the areas over which averages were obtained to plot the ORGs: 0.27°2 (yellow), 0.20°2 (gray), 0.14°2 (red), and 0.07°2 encompassing ≈10 cones (violet). (e) Repeatability of the response: single ORGs (gray dashed) and their mean (solid black) for six repeat trials, in which phase responses were averaged over 0.27°2 for 17.9% bleach. (f) Spatial averaging: ORGs over different areas color-coded according to the rectangles in (d). (g) Maximum intensity projection at COST layer with AO-OCT reveals individual cone photoreceptors. (h) ORGs for a subset of single cones in (g) demonstrating the response in each cone for 0.3% S-cone bleach and 29.7% average L- and M-cone bleach. The magnified view near stimulus onset shows a negligible early response in putative S-cones (blue) compared with L/M cones (orange). (i) ORG early and late response amplitudes for each cone in (g). (j) Histogram of the ORG early and late response magnitude, computed as the Euclidean distance from origin of each data point in (i). The two-component Gaussian mixture model (black dotted line) and its component Gaussians are used to distinguish S-cones (blue fit) from L/M cones (orange fit). The vertical dotted line marks t=0 in (e), (f), and (h) indicating the rising edge of stimulus onset. (a)–(f) are obtained without AO, with 4-mm imaging pupil, at 120-Hz volume rate. (g), (h) are obtained with AO, for 6-mm imaging pupil, at 162-Hz volume rate. The stimulus wavelength for all plots is 528±20  nm. (a)–(j) Adapted from Ref. 268.

Modern medical diagnosis significantly benefits from extracting functional tissue information from structural imaging data (“structure–function correlation”). This is especially important in organs that cannot be biopsied, like the human retina. Retinal function has long been studied with psychophysical methods in humans, e.g., with electrophysiology and electroretinograms.^263^ Non-contact, depth-resolved, optical probing of retinal response to visual stimulation was introduced as optophysiology—an optical analog to electrophysiology. This method takes advantage of the fact that physiological changes in dark-adapted retinas caused by light stimulation can result in local variation of the tissue reflectivity.^264^ At that time, optophysiology could only be demonstrated in isolated rabbit retinas. Ophthalmic OCT technology back then was not sufficiently fast at longer wavelengths performing at sufficiently high sensitivity and resolution to be successfully applied in living animals or humans.^265^ A decade later, light-driven signals of photoreceptors *in vivo* could be measured. Visible light stimulation over a 200-fold intensity range caused correlated rod outer segment (OS) elongation and increased light scattering in wild-type mice, but not in mice lacking the rod G-protein alpha subunit, transducin (Gα(t)), revealing these responses to be triggered by phototransduction.^266^ The diurnal variation in rod OS length in mice was measured using optophysiology, being consistent with prior histological investigations demonstrating that rodent rod discs are phagocytosed by the RPE maximally over several hours around the time of normal light onset. The rate of recovery of rod OSs to baseline length before normal light onset was consistent with the hypothesis that disc membrane synthesis is fairly constant over the diurnal cycle^267^ (Fig. 7).

Fast intrinsic optical signal (IOS), which arises before light-evoked pupillary response, promises to be a unique biomarker for photoreceptor physiology for objective optoretinography with high resolution. In another study, depth-resolved optophysiology verified OS as the anatomic origin of fast photoreceptor-IOS. Dynamic IOS changes were primarily confined at OS boundaries connected with inner segment and RPE, supporting transient OS shrinkage due to phototransduction process as the mechanism of the fast photoreceptor-IOS response.^269^ Non-invasive, objective measurement of light-evoked, functional responses of human rods and cones, measured non-invasively using a synchronized adaptive optics OCT and scanning light ophthalmoscopy system have also been reported recently.^270^ Another recent study revealed that the onset of phototransduction is accompanied by a rapid (<5  ms), nanometer-scale electromechanical deformation in individual human cone photoreceptors. Characterizing this biophysical phenomenon associated with phototransduction *in vivo* was enabled by high-speed phase-resolved optical LFOCT that allowed for sufficient spatiotemporal resolution to visualize the nanometer/millisecond-scale light-induced shape change in photoreceptors.^268^

### 4.3 Visible Light OCT: Unprecedented Axial Resolution and Enhanced OCT Access to Absorption

OCT in the visible wavelength range with unprecedented submicrometer axial resolution achieved by employing a photonic crystal fiber in combination with a sub-15 fs Titanium:sapphire laser was first demonstrated in the beginning of this millennium.^271^^,^^272^ Visible light OCT theoretically provides higher axial resolution than NIR OCT for a given wavelength and bandwidth. To realize this potential in the human retina *in vivo*, the unique technical challenges of visible light OCT must be addressed: incorporating a grating light valve spatial light modulator spectral shaping stage to modify the source spectrum; developing a novel, Fourier transform-free, software axial motion tracking algorithm with fast, magnetically actuated stage to maintain near-optimal axial resolution and sensitivity in the presence of eye motion; and implementing spatially dependent numerical dispersion compensation for the first time in the human eye *in vivo*. Wavelength-dependent images of the outer retina suggest that, beyond merely improving the axial resolution, shorter wavelength visible light may also provide unique advantages for visualizing Bruch’s membrane (BM).^273^ Furthermore, it seems that shorter visible wavelengths improve the visualization of BM in pigmented eyes, where it is located behind a highly scattering layer of melanosomes in the RPE. Monte Carlo simulations of radiative transport suggest that, while absorption and scattering are higher at shorter wavelengths, detected multiply scattered light from the RPE is preferentially attenuated relative to detected backscattered light from BM.^274^

Using visible light OCT, accurate and robust non-invasive measurement of retinal oxygen metabolic rate (${\mathrm{rMRO}}_{2}$) in rat eyes was demonstrated. Both oxygen delivery and ${\mathrm{rMRO}}_{2}$ increased from the highly regulated retinal circulation under hypoxia. The increased oxygen extraction compensated for the deficient oxygen supply from the poorly regulated choroidal circulation. These results have the potential to reveal the fundamental role of oxygen metabolism in various retinal diseases such as age-related macular degeneration (AMD), diabetic retinopathy, and glaucoma.^275^ Oximetry saturation ${\mathrm{sO}}_{2}$ measurements were also recently extended to capillaries and investigated in all three retinal vascular plexuses by amplifying and extracting the spectroscopic signal from each capillary segment under the guidance of OCTA. Using this approach, capillary ${\mathrm{sO}}_{2}$ in the retinal circulation in rats was measured.^276^

**Fig. 8:**
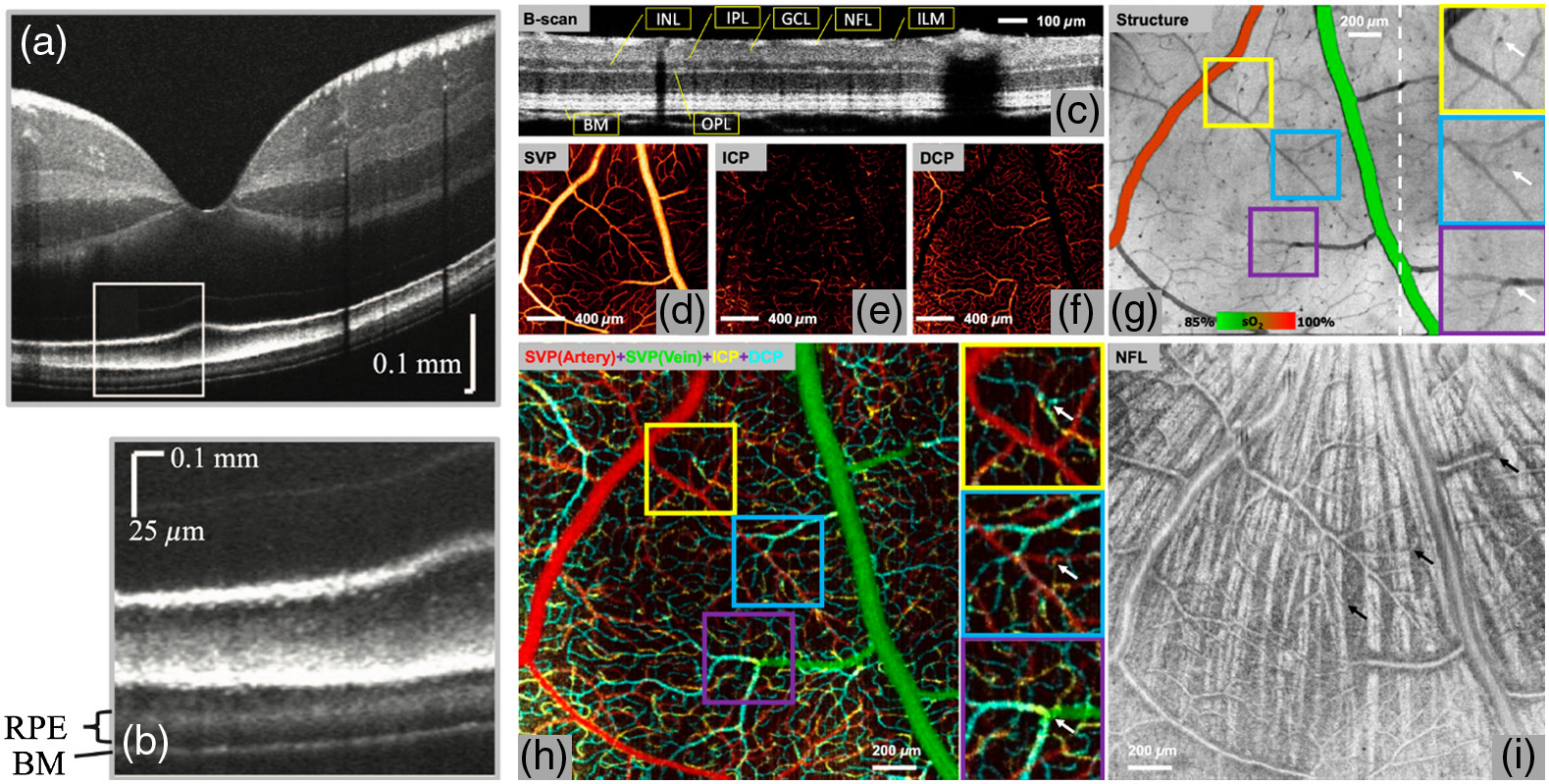
(a)–(b) *In vivo* human retinal visible light OCT; (b) inset from (a). Adapted from Ref. 274. (c) B-scan image of a brown Norway rat retina using visible-light OCT. NFL, nerve fibre layer; GCL, ganglion cell layer; IPL, inner plexiform layer; INL, inner nuclear layer; OPL, outer plexiform layer; and BM, Bruch’s membrane. (d)–(f) *En face* images of vascular/capillary plexuses. SVP projected in the NFL and GCL slabs. ICP projected in the slab containing the inner border of the INL. DCP projected in the slab containing the outer border of the INL. (g) *En face* structural image projected from the ILM to BM, overlaid with measured oxygen saturation (${\mathrm{sO}}_{2}$) values in major vessels to differentiate arteries from veins in an animal breathing 100% $O_{2}$. Interplexus capillaries (white arrows) appear as dark spots due to greater light absorption than neighbouring capillaries. (h) Overlaid *en face* angiograms of three vascular/capillary plexuses to demonstrate the detailed organization of the retinal circulation. Examples of interplexus capillaries (indicated by white arrows in the enlarged images) were validated by observing their presence in corresponding locations. (i) *En face* projection of the NFL slab. The SVP was found to run anterior to the nerve fibre bundles (bright radial striations), which appear posterior to the vessels. The interplexus capillaries (black arrows) penetrate between NFL bundles and connect the SVP to the ICP and DCP. (c)–(i) Adapted from Ref. 276.

*In vivo* depth resolved human imaging of oxygenation in retinal capillaries was recently demonstrated.^277^ In addition, first visible light OCTA with a 100-kHz A-line rate for human retinal imaging was also demonstrated recently, enabling accurate localization of microvasculature down to the capillary level and thus enabling oximetry at vessels <100  μm in diameter. Microvascular hemoglobin oxygen saturation (${\mathrm{sO}}_{2}$) at the feeding and draining vessels at the perifoveal region was demonstrated, allowing for future studies on the role of microvascular oxygen in various retinal pathologies^278^ (Fig. 8).

Another technique enabling OCT access to absorption is photothermal OCT, which monitors changes in the optical path length caused by the photothermal effect. Like in PAI in the photothermal effect, photons are absorbed by chromophores within a sample, causing a localized rise in temperature. Thermoelastic expansion caused by the photothermal effect generates isolated variations in the refractive index, which in turn generates a variation in the optical path length. The optical path length variations typically generated by the photothermal effect, often nanometer in scale, can be resolved with phase-sensitive OCT.^279^ This approach might be extremely attractive for non-destructive 3D molecular imaging deep (∼2  mm) within biological samples with a sensitivity of 14 parts per million (weight/weight) nanoparticles in the sample.^280^ In addition, photothermal OCT could quantify changes in pigmentation that occur in retinal diseases.^281^^,^^282^ *In vivo* photothermal OCT was also demonstrated for cross-sectional in human skin measurement with endogenous absorption agents.^283^ Recently, a swept source dual-wavelength photothermal OCT system was demonstrated for quantitative imaging of microvasculature oxygen saturation and measuring of microvasculature ${\mathrm{sO}}_{2}$ levels in phantom blood vessels with a range of blood flow speeds (0 to 17  mm/s).^284^

### 4.4 Dynamic Contrast OCT

**Fig. 9:**
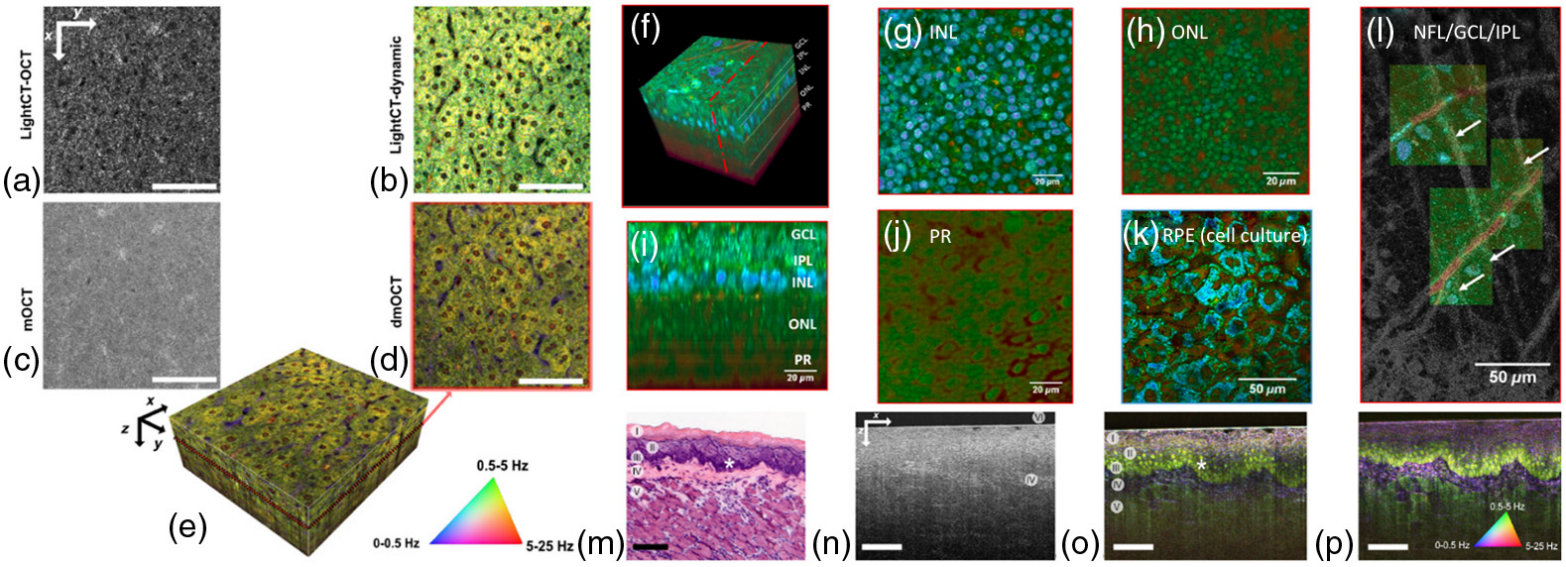
(a) LightCT *en face* OCT image of murine liver. (b) LightCT dynamic OCT *en face* image with corresponding regions. (c) Averaged mOCT *en face* image, which was acquired in the same region. (d) In the corresponding dynamic mOCT image, hepatocytes become visible with nuclei. (e) Cropped volume representation; size: 270⇥285⇥135  μm (xyz); scale bar, 100  μm. (a)–(e) Adapted from Ref. 287. (f) 3D reconstruction of a D-FFOCT image stack in explanted macaque retina over a 120×120  μm FOV. Note that FFOCT signal is damped with increasing penetration depth, so upper retinal layers are more clearly visible than lower ones. *En face* images of (g) inner nuclear layer, (h) outer nuclear layer, and (i) photoreceptor layer presenting a similar appearance to two-photon fluorescence imaging; (j) reconstructed cross section at the location represented by the red dotted line in (f). The cross section in (j) was linearly interpolated to obtain a unitary pixel size ratio. (f) D-FFOCT image of a porcine retinal pigment epithelium cell culture. (g) Overlay of colored D-FFOCT and FFOCT at the interface between the layers of the nerve fibers (white arrows point to nerve bundles that are very bright in static mode and invisible in dynamic mode), ganglion cells (blue and green cells, visible in dynamic mode) and inner plexiform (fibrous network, bottom left, visible in static mode). (f)–(l) Adapted from Ref. 288. (m) HE stained histology of the imaged sample at a different location: (I) cornified layer, (II) granular and spinous layers, (III) basal layer, (IV) lamina propria, (V) muscle, and (VI) glass plate. (n) OCT image of mouse tongue; lamina propria (IV) can be identified by brighter contrast. (o) Corresponding dynamic contrast mOCT image with a focus in the basal cell layer; (I)–(V) and even cell nuclei (*) are visible. (p) Dynamic contrast mOCT image with a focus in the lamina propria; the image size is 380⇥500  μm (zx); scale bar is 100  μm. (m)–(p) Adapted from Ref. 287.

Recently, an extremely powerful new contrast enhancing OCT extension was introduced. It employs a new endogenous approach to reveal subcellular metabolic contrast in fresh *ex vivo* tissues, taking advantage of the time dependence of FF OCT interferometric signals. This method reveals signals linked with local activity of the endogenous scattering elements that can reveal cells where other OCT-based techniques fail or need exogenous contrast agents. Using high-transverse resolution to image intracellular features, the time dependence to identify different dynamics at the millisecond scale on a wide range of organs in normal or pathological conditions is used.^285^ Dynamic contrast OCT has also been applied in freshly excised human esophageal and cervical biopsy samples. Depth-resolved dynamic contrast OCT images of intact tissue show that intracellular dynamics provides a new contrast mechanism that highlights subcellular morphology and activity in epithelial surface maturation patterns^286^ (Fig. 9).

This technique has also been used to combine static, dynamic, and fluorescence contrasts to achieve label-free high-resolution imaging of the living retina and anterior eye with temporal resolution from milliseconds to several hours, allowing for probing biological activity at subcellular scales inside 3D bulk tissue.^288^ By evaluating signal fluctuations, a significant contrast enhancement was demonstrated using TD FFOCT, which makes cellular and subcellular structures visible. The putative cause of the dynamic OCT signal is the site-dependent active motion of cellular structures in the submicrometer range, which provides histology-like contrast.^287^ *In vivo* dynamic contrast OCT was used to quantify layer-resolved microvascular blood flow and volume across the full depth of the mouse neocortex.^289^

Finally, speckle variations induced by intracellular motion in the urothelium were used as a dynamic contrast to segment the urothelium with only two sequential OCT images. This new method opens the possibility of tracking the distribution of the urothelial cells to identify the microinvasion of bladder tumors. This contrast may provide a new mechanism for OCT to diagnose the invasion of urothelial cancerous cells for the better staging of bladder cancer.^290^

Another interesting contrast enhancing OCT extension that gained a lot of attention recently is using the extraction of the optical attenuation coefficient, an important tissue parameter that measures how quickly incident light is attenuated when passing through a medium. Successful extraction of this parameter would facilitate tissue differentiation and enhance the diagnostic value of OCT. With current studies showing attenuation coefficient analysis as a promising technique, further efforts in the development of methods to accurately extract the attenuation coefficient and to explore its potential use for more extensive clinical applications are desired.^291^

### 4.5 Optical Coherence Elastography: Depth Resolved Optical Palpation

**Fig. 10:**
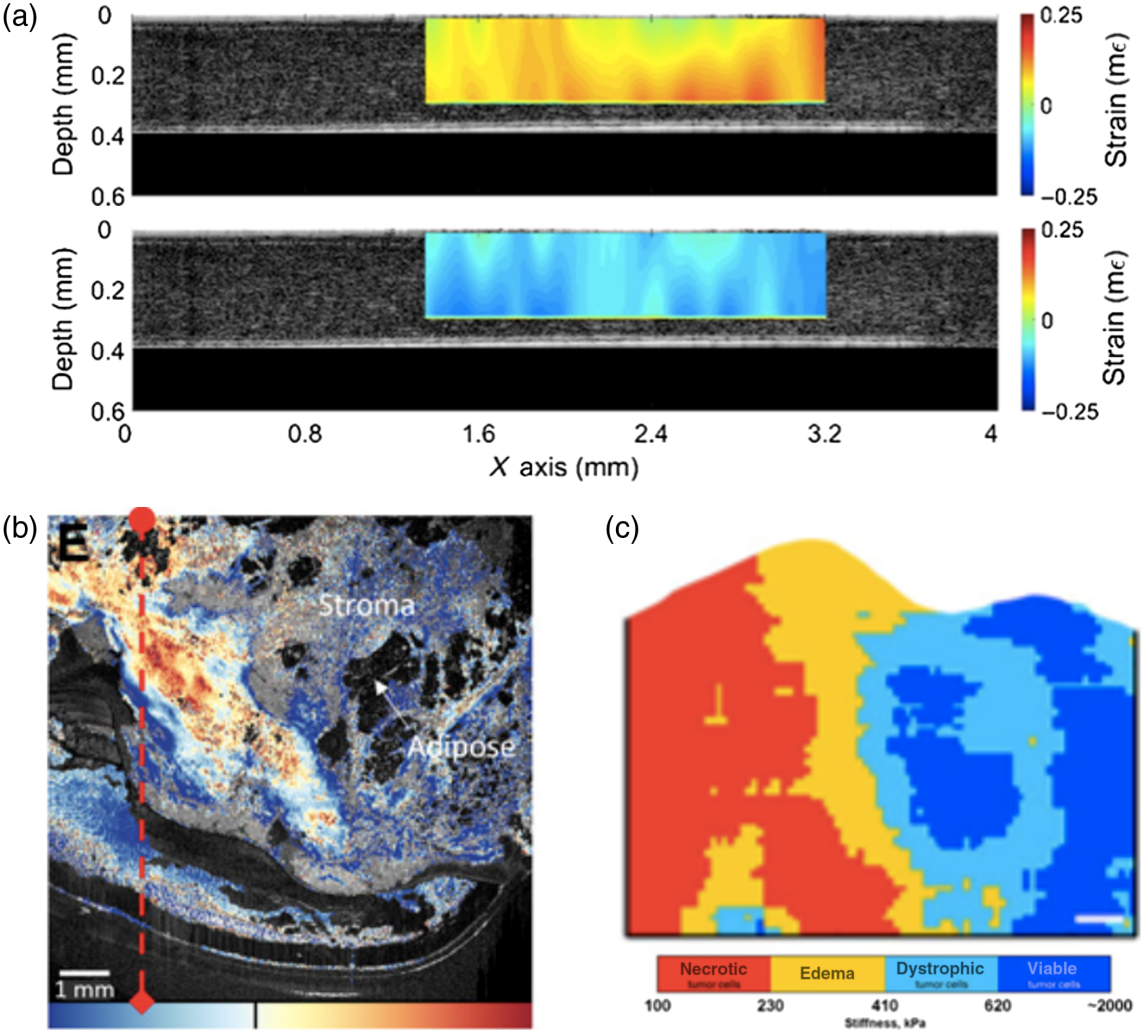
(a) Strain in untreated cornea induced by the heartbeat (orange: compression, blue: relaxation). Adapted from Ref. 307. (b) Qualitative OCE image of invasive ductal carcinoma. Adapted from Ref. 298 with permission from AACR. (c) Automated stiffness-based segmentation of mouse breast cancer *in vivo*. Adapted from Ref. 299.

An extremely promising extension of OCT is OCE. The original idea of elastography is not only to exploit the stiffness changes within soft tissue for diagnosis, much like a physician would during palpation, but also to exactly localize and quantify them. The capability of OCT to give 3D insight into tissue is used to visualize the reaction of a sample to a mechanical force. Multiple methods for deriving this reaction from the OCT data have been presented, e.g., image cross correlation,^292^ feature^293^ or speckle tracking,^294^ optical flow,^295^ combinations^296^ thereof, and phase difference analysis.^297^ From this reaction, the underlying mechanical tissue parameters can be obtained and used for diagnosis. The technology first emerged in US imaging, where systems capable of elastography have been commercially available since the 2000s. Elastography in general is a relative straight forward add-on to OCT, enabling biomechanical contrast at a slightly increased imaging time and data processing complexity. Although a lot of promising approaches and methods utilizing OCE have been published, two major applications are at the moment competing for clinical *in vivo* diagnosis (Fig. 10).

In this sense, the ophthalmic application of OCE is the most advanced, likely because it could build upon decades of OCT experience in this field, which already addressed issues like motion correction and speed. The only novel aspect from an engineering perspective is mechanical loading, which has to be performed cautiously and preferably contactless. Proposed solutions rage from contact shaker excitation,^300^ over microscale^301^ air-pulses,^302^ and acoustic radiation pulses^303^^,^^304^ to heartbeat driven tissue deformation.^301^^,^^305^ De Stefano et al.^306^ demonstrated the feasibility of OCE for identification of keratoconic corneas in humans *in vivo* using a dynamic compressional approach with intensity-based speckle tracking. Since the compression is applied via a motorized stage, direct contact to the cornea is needed, but quantitative analysis also is enabled. In May 2020, Lan et al.^302^ quantified the effects of respiration and heartbeat on patient eye motion as a source of perturbation for OCE *in vivo* and mentioned the idea that this perturbation could also be used as a passive loading method. Only a month later, Nair et al.^305^ gave another indication for the feasibility of non-contact heartbeat ophthalmic OCE in an *ex vivo* porcine eye model with controlled intraocular pressure changes. In January 2021, Nair et al.^307^ achieved heartbeat OCE in an *in vivo* rabbit model, but the method was not completely non-contact as a glass imaging window was used to minimize bulk motion. The heart pulse compressed the corneal tissue against this imaging window, resulting in the desired displacement. OCE data were acquired as multiple 2D B-scans at the same position, which were synchronized with the rabbit’s heartbeat later in postprocessing. The resulting 2D strain maps clearly show the difference between compression and relaxation induced by the pulse. In addition, they were able to differentiate between an untreated cornea and a cornea stiffened via the Dresden protocol.

Although these are already very promising first results, some challenges for clinical *in vivo* patient imaging still remain to be addressed. Since pulse waves travel at a limited speed, they affect different regions in the FOV at different points in time, which can lead to underestimation of the resulting strain and is a challenge for synchronization. For clinical diagnosis, *en face* images and 3D stiffness maps are much more useful than single B-scans. 3D imaging would not only highly increase imaging time but also make the synchronization even more complicated. One also has to be aware that, without the possibility of quantifying the pressure perceived by the investigated tissue, only qualitative OCE results can be expected and quantifying the pressure excited by a heartbeat at every point in the FOV seems impossible at this point in time. However, the potential of ophthalmic OCE for clinical application is huge. The feasibility for diagnosing keratoconus has been shown in the examples above, but also corneal dystrophy or corneal ectasia due to surgical complications are obvious topics in which OCE can deliver fast and uncomplicated diagnosis, as well as in the diagnosis of AMD in which early diagnosis is especially important, as presented by Qian et al.^300^

When it comes to the field of cancer detection and diagnosis, current literature focuses on biopsy images as proof of concept. Not only is cancer often accompanied by significant changes in stiffness, but also the shape of the outlines of cancer has diagnostic potential.^308^ A good example is given by Kennedy et al.,^298^ who showed that cancer margin detection with OCE yields a 24% higher sensitivity than with OCT only. Multiple tumor types have been investigated, and OCE proved to be an accurate predictor of malignancy for all of them. A phase-sensitive, quasi-static compression OCE system was used.^309^

The future of OCE lies in intraoperative *in vivo* imaging for determining tumor margins of cancerous tissue to be removed, requiring not only high-speed OCT imaging to minimize the influence of motion artifacts but also high-speed processing to enable real-time view. Depending on the chosen biomechanical model and processing approach, this task requires expensive hardware and well-versed software implementation. Another challenge to keep in mind is OCE’s vulnerability to laser and system instabilities, which are even more difficult to control in a surgical environment.

Nonetheless, surgical applications of OCE are close. Plekhanov et al.^299^ presented a phase-sensitive, quasi-static compressional OCE method that is capable of segmenting cancerous regions in a mouse model *in vivo*. They were able to differentiate between viable, dystrophic, and necrotic tumor cells as well as edema zones and validated their findings via comparison with histological results. A 9-day study, comparing the effect of two different chemotherapeutics with untreated control, was performed as well. In a similar study,^310^ the combination of OCE with OCTA was shown to be an easy way to add functional information, improving the quality of chemotherapeutic treatment efficiency studies.

As can be seen in the aforementioned examples, quasi-static compression OCE, mostly phase-sensitive with some sort of compliance layer as stress sensor, is currently the most advanced OCE method. In the last few years, other approaches rose to the horizon of feasibility, e.g., vibrational OCE,^311^^,^^312^ which uses OCT to record the reaction of a sample to a variation of vibration frequencies. With this, a 3D resonant frequency map of the sample can be generated and viscoelastic behavior can be investigated. Another proposed application, although more focused on usage in studies than for clinical diagnosis, is in intravascular OCE.^313^^,^^314^ Since atherosclerosis is the leading cause of human death worldwide, continued effort is put into understanding the generation, buildup, and rupture of intravascular plaques.

Overall OCE has come far, and routine clinical usage especially in the ophthalmic field is to be expected in the next few years. The next steps to this goal are achieving faster imaging and data processing and even better motion correction. Although some OCE experts and clinicians argue that there is no need for quantifying tissue stiffness, as long as there is qualitative biomechanical contrast, in the long term it might make a lot of clinical sense to find a quantitative stiffness atlas, which allows for diagnostic guidance.

## 5 OCT and Deep Learning, Neural Networks, and Artificial Intelligence

Since its introduction in 1959, AI technology has evolved rapidly and helped benefit research, industry, and medicine. The simultaneous maturation of multiple digital and telecommunications technologies in 2020 has created an unprecedented opportunity for ophthalmology to adapt to new models of care using telehealth supported by digital innovations. These digital innovations include AI, 5th generation (5G) telecommunication networks, and the Internet of Things (IoT), which create an interdependent ecosystem offering opportunities to develop new models of eye care addressing the challenges of COVID-19 and beyond. Deep learning, as a process of AI, is used in radiology, ophthalmology, and in increasingly more other medical fields for data analysis, segmentation, automated diagnosis, and possible prognosis. The association of deep learning and OCT technologies has proven reliable, and about 400 papers have been published in this field, with more than half of them published in the last 3 years.

**Fig. 11:**
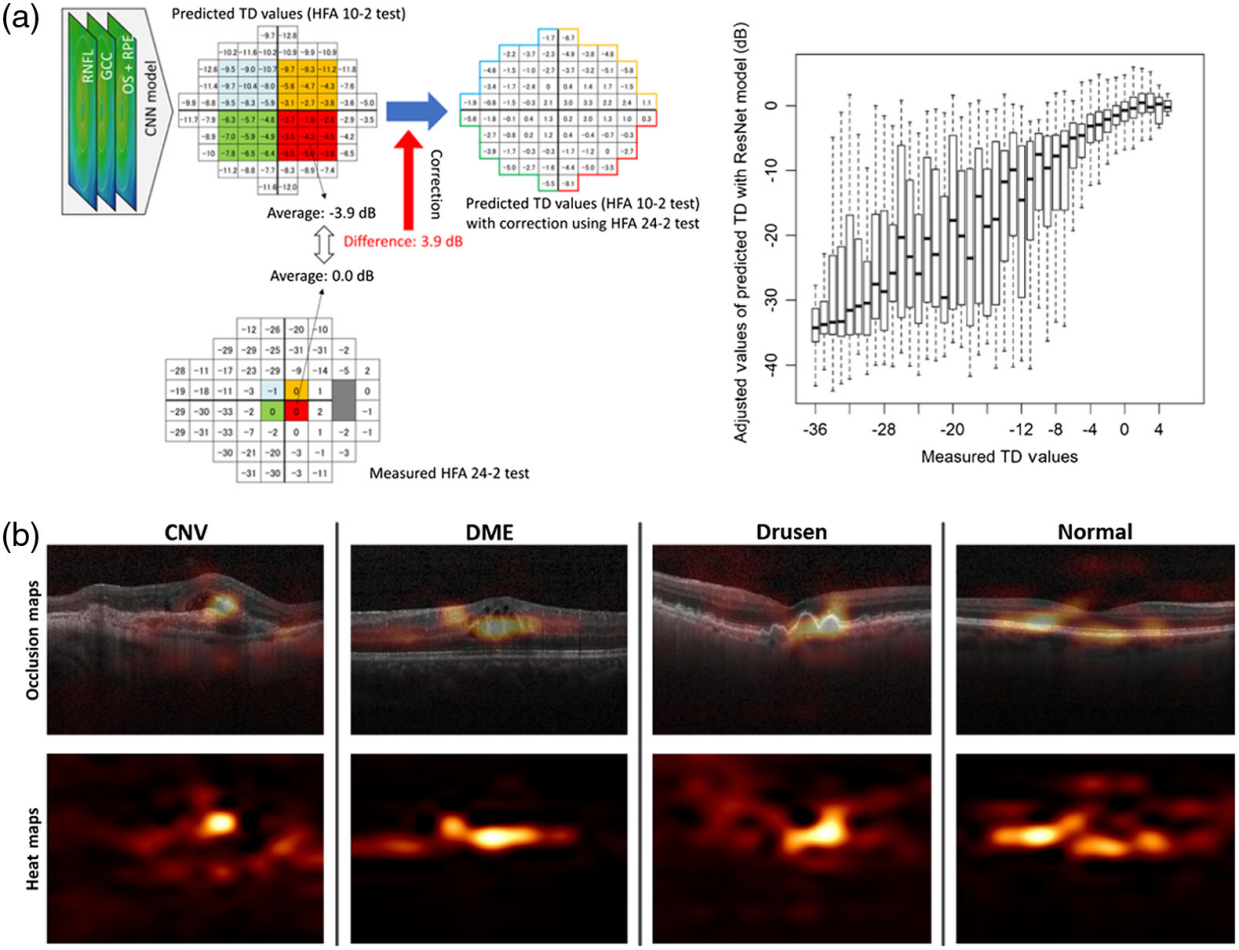
(a) A CNN model was used to predict TD values in the central 10 deg of the visual field (corresponding to HFA 10-2 test data). The mean TD values within the central 6 deg at each quadrant (36 and 4 test points for the HFA 10-2 and HFA 24-2 tests, respectively) were calculated. The corresponding test grids in the inferonasal quadrant are shown. The differences between these values were calculated, and the predicted TD values were adjusted as per the calculated differences in each sector. CNN, convolutional neural network; GCC, ganglion cell complex; HFA, Humphrey field analyzer; OS, outer segment; RNFL, retinal nerve fiber layer; RPE, retinal pigment epithelium; and TD, total deviation. The relationship between the actual TD values and the predicted TD values using the ResNet model adjusted with the measured TD values corresponding to the innermost four points of the HFA 24-2 test. HFA, Humphrey field analyzer and TD, total deviation. Adapted from Ref. 319. (b) Occlusion testing maps showing most significant regions for detecting retinal diseases. In these images, golden regions indicate a large impact on model predictions while orange and red regions indicate a very limited impact on predictions. The heat map was created after prediction by assigning the softmax probability of the correct label to each occluded area. The occlusion map was generated by superimposing the heat map on the input image. CNV, choroidal neovascularization and DME, diabetic macular edema. Adapted from Ref. 318.

Ophthalmology has thrived in some of these areas partly due to its many image-based investigations. Telehealth and AI provide synchronous solutions to challenges faced by ophthalmologists and healthcare providers worldwide. AI definitely has potential and will be part of the decision-making progress regarding the scientific investigation, diagnosis, and therapeutic management. Hence, AI-enhanced OCT has recently been used for the detection of retinal diseases and improving the diagnostic performance of the eye’s posterior segment diseases^315^^–^^318^ (Fig. 11).

In addition to AI-enhanced OCT application enabling improved OCT image quality or enhancing OCT segmentation, functional prediction of organs from morphological OCT data will be of significant future clinical interest.^319^

Several recent studies have reported high-diagnostic performances of AI models. However, significant methodological challenges still exist in applying these models in real-world clinical practice. Lack of large image datasets from multiple OCT devices, non-standardized imaging or postprocessing protocols between devices, limited graphics processing unit capabilities for exploiting 3D features, and inconsistency in the reporting metrics are major hurdles in enabling AI for OCT analyses.^320^^,^^321^ Furthermore, machine learning and AI for health must be reproducible to ensure reliable clinical use. Recent evaluations found that machine learning for health fared poorly compared with other areas regarding reproducibility metrics, such as dataset and code accessibility.^322^^,^^323^

## 6 Miniaturized, Cost-Effective, and Portable OCT: OCT on a Chip, Home-OCT, and Self-OCT

Most commercial OCT devices currently have a footprint of $∼1^{2}\;m$ and cost up to USD 180.000, which usually inhibits its widespread availability and limits usage of OCT to clinics or large ophthalmic practices.^324^

In a typical diagnostic ophthalmic OCT setting, the patient has to remain in an upright position with his head placed on a chin rest and fixating on a target. Certain retinal diseases such as AMD, glaucoma, or diabetic retinopathy require a regular screening to monitor the disease and schedule or monitor therapy. These diseases normally affect elderly immobile or even bedridden patients for whom going to the hospital or practices for OCT diagnosis is challenging. For diagnosis of retinopathy of prematurity, a disease affecting newborn premature infants as well as for younger children in general, current commercial OCT devices are also not suitable. The same holds for space-related neuro-ocular syndrome describing ocular pathological changes that occur during space flight and exposure to microgravity, respectively.^325^ Commercially available ophthalmic OCT technology has been used in the past, but it is too bulky and time-consuming to be used in outer space.

**Fig. 12:**
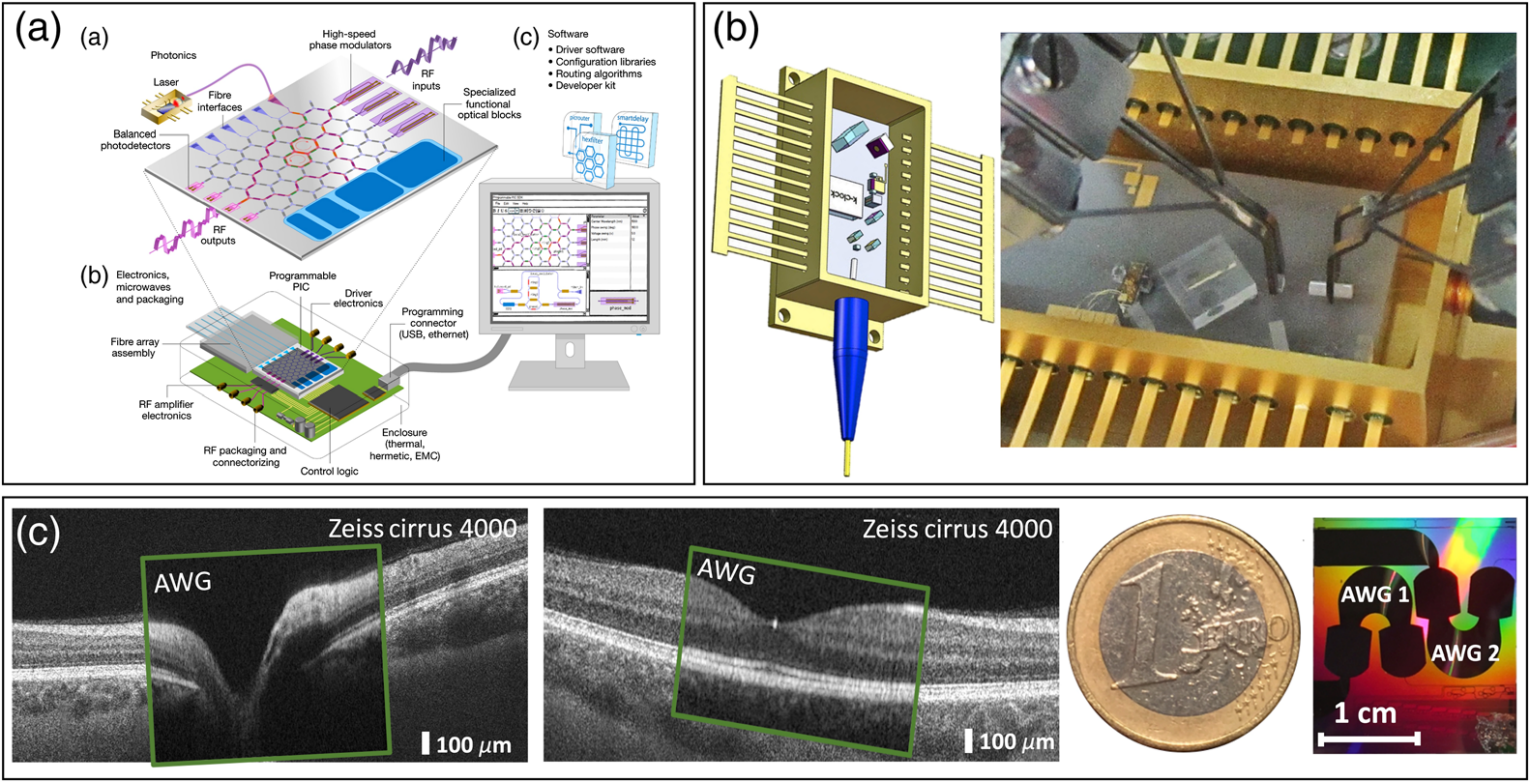
Overview of most promising future OCT technologies: (a) schematic of a programmable PIC with several functional layers: a programmable mesh of photonic gates, phase modulators, and detectors packaged with electronic AD drivers that are connected to a computer with which the user can manipulate and access the photonic functionality. Reprinted from Ref. 327 with permission from Springer Nature. (b) Three-dimensional model of a swept laser and robotic placement of microcomponents using micro-optics in a 14-pin butterfly package. Reprinted from Ref. 328 (c) First *in vivo* human retinal imaging using an on-chip grating (arrayed waveguide grating, AWG)-based SD-OCT. Two AWGs are shown in the used PIC, which measures only 2×2  cm. Adapted from Ref. 329.

Hence, light weighted, cost-effective, and compact (ideally handheld) OCT devices would be required for early diagnosis and proper therapy management of all of the abovementioned retinal diseases. It is noteworthy that recent market reports predict (for the first time in such reports) separate compound annual growth rates for handheld and integrated OCT systems. Interestingly, compound annual growth rates (CAGR) is 8.9% for the next 7 years despite COVID-19 and for OCT in ophthalmology globally about 5% to 7% depending on the region.^326^ In addition, “home-OCT” or “self-OCT” are terms that have evolved to describe the need for a compact, easy to use, and cost-effective OCT device that could be used unsupervised by patients at home or astronauts in space. The term “miniaturized OCT” therefore describes more than solely the reduction of size but also a reduction of costs. An increase of flexibility of OCT devices is ultimately a highly desirable goal for extending the application and availability of OCT and exploring potential new markets for OCT. Point-of-care diagnostics, home-based disease monitoring, extension of medical care in third-world countries and low-recourse settings, and even extra-terrestrial health care are application fields that would benefit from a compact, cost-effective, mobile, handheld, and even patient operated OCT system (Fig. 12).

Beyond ophthalmologic care, there is a strong need for flexible and compact OCT devices as well. Dermatologic care, oral cavity, or inner ear imaging are only a few examples of OCT applications that require at least a flexible (handheld) probe. As a broad screening device, a general practitioner might benefit from an OCT device that is capable of imaging several areas of the human body. This could be achieved by exchangeable sample arm adapters as recently proposed,^330^ a system capable of imaging ophthalmic, inner ear, and other tissues by exchangeable sample arm adapters.

Currently, there are three promising approaches to miniaturizing OCT.

1. The development of flexible handheld probes for OCT, being motivated by immobile patients or simply the anatomical nature of the sample location. To date, they come with a mobile cart with a footprint of $∼1\;m^{2}$.
2. The use of already available off-the-shelf micro-optical components to miniaturize OCT systems by smart and compact packaging.
3. Finally, PIC as a platform that has its origin in telecommunications. Using the same fabrication plants as those for CMOS electronics to grow these chips, PICs guide and manipulate light just as commonly used fiber-based systems but can be produced at a significant smaller size as well as costs in addition to be maintenance free.

The need for a mobile OCT device has been partly met by industry as devices are equipped with mobile carts, which permit mobility within the clinics. Some companies offer handheld probe solutions for more flexibility in terms of bringing the scanning probe to the patient.^331^^–^^333^ However, these systems are still rather bulky, the handheld probes are fairly heavy (1.5 to 2.2 kg), and the weight of these systems (∼30  kg) challenge easy mobility but especially portability.

Demonstrations of (partly) miniaturized OCT systems in research have been accomplished in ophthalmic care using handheld probes,^330^^,^^334^^–^^343^ off-the-shelf micro-optical components^52^^,^^334^^,^^344^ or PICs^329^^,^^345^^,^^346^ for dermatologic care using handheld probes,^347^^–^^353^ compact packaging of off-the-shelf components,^354^ PICs,^353^^,^^355^^–^^357^ and handheld probes for oral^358^ and for inner ear^359^^–^^362^ imaging. The usage of off-the-shelf microcomponents seems to be the most mature technique for miniaturized OCT at the moment. Very compact and mobile OCT systems with acceptable imaging performance have been accomplished.^334^^,^^344^^,^^354^

Historically, many medical applications have benefited from the development of components for telecom and entertainment applications, respectively. The miniaturization of OCT has benefited from these developments as well. MEMS mirrors were originally developed in smart phones and CCDs that were produced for multimedia, smartphones, and entertainment; they are components that are produced at scale and can be adapted to the needs of OCT applications. Likewise, the telecom-driven development of PICs supports the miniaturization of OCT. With the increasing need for more powerful telecom and entertainment devices, the industry invested in alternatives for electronical data transfer and manipulation. This technology is still in its infancy, and the first *in vivo* human retinal imaging was shown in Fig. 12(c).^329^ Another example is a battery driven, tablet-like OCT system for dermatologic application that has been commercialized.^355^ These are promising results for the future of miniaturized OCT using PICs.

In addition to its small form factor, multiple functional photonic building blocks can be printed on a single chip, which opens up new and/or easier realizable possibilities for OCT system designs, such as multichannel sample arm configuration/parallelized imaging as proposed recently.^363^ Several sample arm paths could scan the sample in parallel, which would increase the effective A-scan rate without sacrificing imaging performance.

Detection and postprocessing electronics can be co-integrated on the same chip, retaining a small form factor of the full OCT engine. Optical FPGAs, i.e., programmable PICs [Fig. 12(a)], are an exciting research field that could establish a universal OCT engine, programmable to specific needs of an OCT system.^327^

With the outbreak of the Covid-19 pandemic in 2020 and the need to reduce social interaction, non-urgent medical appointments were cancelled and postponed. To counteract the reduced medical care, the use of telemedicine was rapidly incorporated, which reduced costs and was more effective and therefore has the potential to become the “new normal.”^316^^,^^364^ Miniaturized, low-cost, mobile, and automated OCT will play a significant role in the successful adaption toward increased use and acceptance of telemedicine.

However, there are many aspects that need to be considered and solved, regardless of the technique used to miniaturize OCT. Weight and size have to be light and small enough for a patient to transport. The device needs to be simple and easy to use, comfortable for the patient, effectively performing, and secure. A secure data transfer has to be ensured and a timely diagnosis/feedback to the patient has to be guaranteed for optimum willingness to use the device. Audio instructions, automated sample alignment, and optimized ergonomic design can help increase the patient compliance. AI^365^^,^^366^ and cloud-based computing^367^ are promising platforms to increase regular monitoring and simultaneously reduce the need for computationally intensive and expensive equipment. At the same time, these might relax power consumption of the device, which could enable battery driven devices, which in turn increases flexibility and mobility even further.

In conclusion, the technology of miniaturized OCT is still young and foreshadows an exciting future of more widespread, mainstream, automated, intelligent, and smart point-of-care devices for early detection of pathological changes.

## 7 Clinical Translation of Optical Coherence Tomography

OCT has revolutionized ophthalmology over the past two to three decades. Its unique ability to resolve the retina’s layered structure was a gamechanger for diagnosing many retinal diseases and monitoring their treatment. Although clinical benefit could also be demonstrated early on in other medical fields such as cardiology, dermatology, and endoscopy, its widespread adoption in these fields is still lacking. This is mostly due to OCT’s lower benefit/cost ratio in these fields and competition with other, often much cheaper, imaging modalities. With OCT becoming cheaper over time, enabled by technological advancements like photonic integration and the availability of cheaper swept-source lasers, we expect OCT also to become more prevalent in the clinical routine in many fields outside ophthalmology and outside the traditional clinical setting. AI algorithms for image quality enhancements, self-alignment, and clinical decision support will enable a new level of usability, much more comparable to consumer electronic devices than sophisticated imaging devices that required expert operators. This will pave the way for OCT systems out of medical specialist’s offices to general practitioners, pharmacies, and even patients’ homes. This trend has the potential to lower health care costs in developed countries but is foremost going to have a huge impact on the quality of life of immobile patients, patients in rural regions, or low-resource countries.

At the same time, we expect OCT’s performance and application to continue to expand at a rapid rate. OCTA was most likely only the first functional extension that boosted the clinical value of OCT. MEMS tunable VCSELs are going to enable commercial OCT systems at hundreds of kHz to MHz A-scan rates. Such speeds favor additional functional methods *in vivo*, such as, for example, the non-invasive, objective measurement of retinal function. MHz speeds are further going to enable the live observation of dynamic processes, making it a great visualization tool for ophthalmic surgery. Such a comprehensive live 3D view of the surgical scene will help in developing robot assisted surgery and will eventually even make autonomous robotic surgery a reality.

Once the “speed-limitations” of single-point scanning OCT are reached, we expect a shift to parallel OCT configurations. LFOCT has the potential to become the implementation of choice due to its advantages with respect to laser safety, ability to suppress multiply scattered light, and availability of components.

Endoscopic OCT may be another area that will greatly benefit from the miniaturization of OCT. It will eventually allow for movement away from traditional endoscopes to self-contained capsular devices that will be swallowed by the patient and collect and transmit data to external devices autonomously. On the other hand, the combination of OCT with other imaging modalities still has to prove its clinical added value to justify the increased complexity and cost. Closest to clinical translation might be the combination of OCT with complementary spectroscopy methods such as infrared spectroscopy used in intravascular probes or RS.

Fast translation of new developments to their commercial exploitation and ultimately to the benefit of patients critically depends on the regulatory ecosystem.

### 7.1 Medical Device Regulations

Europe currently sees the transition from the medical device directives to the medical device regulation as a need to be implemented as national law by each member state. They provide a tight framework not only for medical device industries but also for academic institutions, affecting research and development of new technologies already at its very roots. Clearly, regulatory approval is essentially needed for safe applications on human beings. Academic entities are therefore now challenged to allocate sufficient resources and to build supporting structures to enable internationally competitive research to harmonize the high flexibility needed at early research stages with regulatory demands. Novel technologies need to be convincing enough for industry to pick them up for further development, which needs already substantive validation in a clinical setting with all of the required regulatory hurdles. Only with proper support can a widening of the “valley of depth” be avoided.

### 7.2 Standardization

A translation of promising OCT technologies to market standardization is an important topic as discussed by Waterhouse et al.^368^ For example, the question of appropriate laser power levels for internal organ tissue is not yet covered by laser safety standards. The current assumption is that all internal organs respond similar to the laser exposure. However, already the tissue texture leaves the impression of major differences between the tissue types. With a general standardization on given performance parameters, for instance, the relation to an appropriate standard would be possible at an early stage of translational research. This aims furthermore at the repeatability and reproducibility within multicentral clinical trials. Assessment of performance parameters at an early development stage is difficult because little or no validated *ex vivo* models are present to benchmark performances of a newly developed device. Most of these arguments merge into the argument of cost-effectiveness during research and development. Having standardized benchmarking steps and milestones alongside the development processes in academia or industry may save resources. Translational research actually is facing more than one “valley of death:” the first one arises from translating laboratory systems and findings to the patient bedside and the second one occurs during the attempt to bring the gained knowledge into clinical practice and health decision-making procedures.^369^ With clear standards for benchmarking, there might be a higher chance for seeding product development and streamlining translational research to finally appear next to the patient.

## 8 Conclusion

OCT has existed for 30 years and is definitely here to stay, to keep scientists and engineers busy, and to significantly support clinicians and life scientists in their daily routine work. It is absolutely noteworthy that OCT has not been completely exploited and has considerable growth potential. This is especially important for younger scientists choosing their scientific topic and academic or industrial career field. From a scientific point of view, the last three decades have shown a continuous increase of scientific output. With novel disruptive technologies on the horizon perfectly matching those needed by OCT, it is very unlikely that this will change in the near future. Extrapolating publishing performance of the last 20 years, a saturation of yearly publication output at very high level of about 9500 can be expected in around 10 years from now—if ever. From an industrial point of view, recent market reports indicate a global market perspective of about USD 1.5 billion in 2023.^370^ It is noteworthy that these recent market reports predict (for the first time in such reports) separate compound annual growth rates for handheld and integrated OCT system. Interestingly, CAGR is 8.9% for the next 7 years despite COVID-19 and for OCT in ophthalmology globally about 5% to 7% depending on the region.^326^ As of now, miniaturized OCT is one of the most prominent OCT market trends picking up pace in global industry. Rising interest in miniature, low-cost portable OCT indicates the huge opportunity in the years to come, with miniaturization and improvements in device designing and packaging currently being the key focus areas. As a result, it is expected that the handheld OCT devices segment can make significant progress in the years ahead, entering new larger unexploited markets for OCT.

Another important key-technological prerequisite for not only miniaturized, portable OCT but also for nearly all other OCT applications is imaging speed. Cost-effective swept source laser technology and/or efficient parallelized scanning schemes (multiple single beams, line-field, and full-field) will enable OCT A-scan rates beyond 1 MHz in the very near future as a standard OCT specification. This will be especially essential for handheld OCT to avoid motion artifacts, will enable large FOVs (e.g., wide-field OCT and OCTA, high-speed catheters/capsules), will permit the detection of fast signals (e.g., IOSs), and will foster 4D OCT for intraoperative, surgical guidance or high-speed imaging in life sciences. It will also enable proper sampling, especially with increasing FOVs and improved transverse OCT resolution. With the recent successful initiation of visible light OCT, the future will not reveal significantly new additional wavelength regions for OCT. The UV and MID-IR regions are theoretically interesting in terms of resolution and absorption but are inherently challenging in terms of light sources, optics, and detection and hence may not be widely translated to (bio)medical and clinical applications.

The momentary successful trend in clinical imaging to combine complementary imaging modalities to get “the best of both/all worlds” will—regarding multimodal OCT setups—continue and significantly increase—not only at the microscopy level but also at the endoscopy/optical needle level. Multimodal optical imaging incorporating OCT will especially compensate for the deficits of OCT (metabolism, molecular sensitivity, penetration depth, and loss of contrast). OCT will act like a GPS by prescreening the tissue at a wide FOV with microscopic resolution and then other techniques will zoom in at the subcellular or molecular level for enabling morpho-molecular or morpho-metabolic tissue information. OCT’s top (bio)medical application will stay in ophthalmology with cardiology following. Oncologic diagnosis in different organs (e.g., skin, GI tract, and others) will increase due to multimodal imaging approaches. The future will also see improved microscopic OCT performance due to speed, multimodality, and enhanced contrast. This improved microscopic OCT performance will also foster intraoperative OCT guidance—especially in ophthalmology and neurosurgery. On the other hand, a lack of favorable medical reimbursement schemes and limited clinical data are top deterrents in the global market for OCT.

From a technological perspective, SD OCT will, for the near future, stay dominant mainly in the 800-nm wavelength region (in addition to the visible light OCT segment) because of the availability of mature cost-effective light source and detector technology—enabling high resolution with moderate optical bandwidths and good contrast. From a perspective point of view SS OCT should be the dominant choice for years. This will only take place if easy to use, cost-effective swept sources that do not shift complexity into detection will be available.

Functional technologies are always much more challenging than pure morphology-based techniques. Successful future contrast enhancing OCT extensions must be technologically simple and easy to interpret and must have significant realistic clinical impact. OCTA is an exquisite example of providing label-free perfusion information acting as a non-invasive angiography technique. Manual palpation is one of the first diagnostic techniques in mankind—hence OCE might be successful in the near future as an optical analog. When it comes to correlated tissue structure and function optophysiology/optoretinography has huge diagnostic potential due to the retina being easily optically accessible, performing non-invasive detection of IOSs, and providing access to the brain. The future might also enable the establishment of new diagnostic biomarkers empowered by depth resolved tissue contrast due to dynamic contrast OCT or quantification of tissue attenuation.

With either, the future of OCT and in general of biophotonics is bright due to numerous upcoming disruptive cost-effective technologies that will enable industry and academia to continuously improve the performance and hence diagnostic capability of existing optical techniques or to establish new ones.
